# Empowered pro-inflammatory features in Ly6C monocytes and altered antigen-presenting capacity in Ly6C^high^ monocytes in diabetic mice

**DOI:** 10.3389/fimmu.2026.1794704

**Published:** 2026-05-08

**Authors:** Pingping Yang, Pu Fang, Lizhe Sun, Lu Liu, Linghua Fu, Hang Xi, Xianwei Wang, Rihab Bouchareb, Daqing Zhang, Qinghua Wu, Yong Ji, Xiaofeng Yang, Hong Wang

**Affiliations:** 1 Department of Endocrinology and Metabolism, The Second Affiliated Hospital, Jiangxi Medical College, Nanchang University, Nanchang, Jiangxi, China; 2 Center for Metabolic Disease Research, Department of Cardiovascular Science, Lewis Kats School of Medicine, Temple University, Philadelphia, PA, United States; 3 Department of Cardiovascular Medicine, The First Affiliated Hospital of Xi’an Jiaotong University, Xi’an, China; 4 Departments of Cardiovascular Medicine, The Second Affiliated Hospital, Jiangxi Medical College, Nanchang University, Nanchang, Jiangxi, China; 5 Department of Cardiology, Shengjing Hospital of China Medical University, Shenyang, China; 6 State Key Laboratory of Frigid Zone Cardiovascular Diseases (SKLFZCD), Harbin Medical University, Harbin, Heilongjiang, China

**Keywords:** cytokine, diabetes, immune checkpoint, inflammatory, Ly6C monocyte

## Abstract

**Rationale:**

Inflammatory monocyte (MC) subset polarization is a hallmark of systemic and tissue inflammatory feature in diabetes. The underlying molecular mechanism remains unclear.

**Methods and results:**

Blood pro-inflammatory Ly6C^high^ and anti-inflammatory Ly6C^low^ MC subsets were isolated from control (C57/BL6), type 1 diabetes mellitus (T1DM), and type 2 diabetes mellitus (T2DM) mice by flow cytometry sorting and subjected to bulk high-throughput RNA-sequencing. Intensive and integrative functional bioinformatic studies were performed by analyzing the transcriptome through seven pairs of comparison between Ly6C^high^ and Ly6C^low^ MC subsets and between different mouse groups. We examined molecular features of three key immune activation signals in innate and adaptive immune responses, including signal 1 antigen (Ag)-presenting, signal 2 immune checkpoint, and signal 3 cytokine, and their upstream transcription factors (TF). A total of 10 differentially expressed genes (DEG) of MHC-II molecules (signal 1) presented a low expression profile in Ly6C^high^ MCs from all mice and mostly further reduced in T2DM Ly6C^high^ MC. Ly6C^high^ MCs show high intercellular inflammatory propagation based on immune checkpoint/cytokine-ligand expression but low intracellular inflammatory capacity based on immune checkpoint/cytokine-receptor expression, both further enhanced in T1/T2DM. In contrast, Ly6C^low^ MCs exhibit low intercellular propagation but high intracellular inflammatory capacity; both also increased in T1/T2DM. Furthermore, 921 upstream DEG transcription factors and 17 transcriptional axes were recognized in diabetic Ly6C^high^ MCs by IPA upstream regulator analysis. Finally, three critical transcriptional axes that drove the altered immunological phenotype in Ly6C^high^ MCs were recognized: ↓PAX5-↓CIITA-↓Cd74/H2-Eb2 for reduced Ag-presenting power, ↓MYC-↑Sema4a for elevated intercellular inflammatory propagation capacity, and ↑CEBPA/E-↑Csf2ra/3r for enhanced intracellular inflammatory propagation capacity in MC.

**Conclusions:**

We have three major discoveries: 1) Ly6C^high^ MCs exhibit lower Ag-presenting power, which was further suppressed in T2DM, mostly via ↓PAX5-↓CIITA-↓CD74 regulation. 2) Ly6C^high^ MCs display high intercellular inflammatory propagation but low intracellular inflammatory capacity, both further enhanced in T1/T2DM, mostly via ↓MYC-↑Sema4a and ↑CEBPA/E-↑Csf2ra/3r regulation. 3) Ly6C^low^ MCs acquired additional intercellular and intracellular inflammatory capacity in T1/T2DM.

## Graphical Abstract

**Figure:**
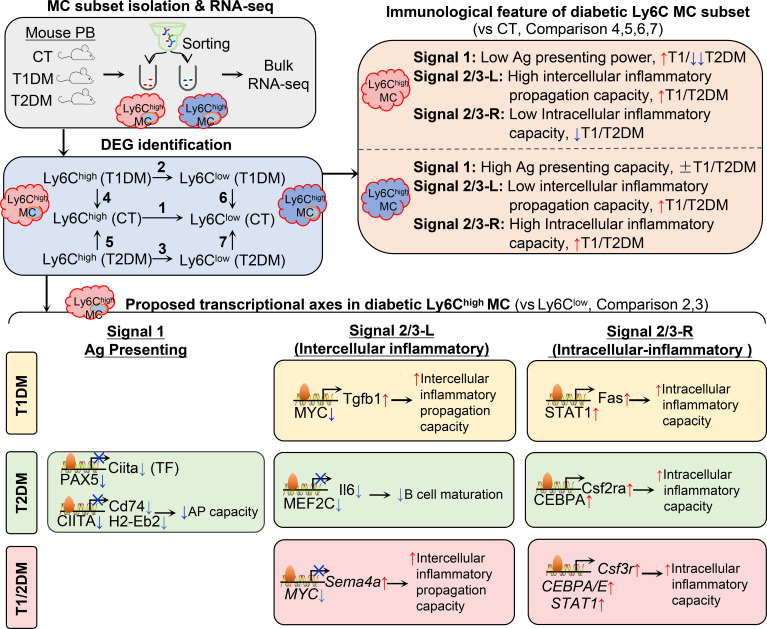
.RNA-Seq were performed in Ly6C^high^ and Ly6C^low^ MC isolated by flow cytometry sorting from peripheral blood of CT, T1DM (STZ, *ip*) and T2DM (*db/db*) mice. DEG in Ly6C MC subset were identified via seven pairs of comparisons between transcriptome from MC subset and mouse groups. Immunological feature of diabetic Ly6C MC subset are molded based on gene expression changes in three classical Immunol signaling (AP, immune checkpoint, cytokines) by comparing each subsets with that in the control mice. Major transcriptional axes in diabetic Ly6C^high^ MC are molded based on TF DEG factionally matched with target immunological DEG identified by comparing with that in Ly6C^low^ in the same mice.

## Introduction

Monocytes (MCs) are the largest type of leukocyte in the blood, accounting for 3%-8% of the blood leukocytes, and a crucial constituent of the innate immunity (1). MCs play essential roles in the first line of defense against pathogens and in the initiation of adaptive immune responses (2). MCs can be classified into multiple subsets each with defined specialized function (3, 4). Mouse MC subsets are classified based on the surface expression of the lymphocyte antigen 6 complex locus C (Ly6C), a mouse-specific glycosylphosphatidylinositol-anchored cell surface protein predominantly expressed on subsets of myeloid cells and does not have a direct homolog in humans (4, 5), whereas human MCs are separated based on their surface expression of CD14 and CD16. The mouse Ly6C^high^ MC subset is also termed as classical MC, similar to human CD14^++^CD16^+^ intermediate MC, processing pro-inflammatory function (4). The mouse Ly6C^low^ MC subset is described as non-classical MC, similar to human CD14^+^CD16^++^ non-classical MC, which plays a vital role in homeostasis and patrolling (patrol blood vessels and accumulate at inflammatory sites to remove debris) (4). We proposed to use cluster of differentiation 40 (CD40) as a novel molecular marker to define human MC subsets (6). CD40 is a stimulatory immune checkpoint receptor expressed in myeloid cells and other antigen (Ag)-presenting cells which play a broad role in various immunological processes contributing to both humoral and cell-mediated immune responses (7). The CD40^+^CD14^+^ MCs have a stronger inflammatory feature than that in the inflammatory intermediate CD14^++^CD16^+^ MC, which correlated with the severity of chronic kidney disease (CKD) stages (7, 8). Ly6C has been continually used to define the inflammatory function of the mouse MC subset in recently emerging single-cell RNA sequencing (scRNA-Seq) studies (9). In addition, other molecules were considered as alternative segregation for MC subsets. For example, mouse Tie-2^+^CD11b^+^CD115^+^ MCs were suggested for pro-angiogenic properties and wound healing (10). Human HLA-DR^low^S100A^high^CD45^+^ MCs were correlated with immunosuppressive function and may be responsible for sepsis-induced immune depression (11).

Accumulated evidence has shown that MC subsets are implicated in the pathogenesis of diabetes. Inflammatory MCs and macrophage (Mϕ) subsets were found increased in human and mouse type 1 diabetes mellitus (T1DM) (12). Ly6C^high^ MCs were related with pro-inflammatory and fibrotic function, and the transformation of the Ly6C^high^ to Ly6C^low^ phenotype was delayed in diabetes wounds in high fat diet-induced type 2 diabetes mellitus (T2DM) mice (13). Moreover, inflammatory MC subset MC4 (CD14^+^CD16^+^HLADR^+^KLRD1^+^PRF1^+^ NK-like) was found increased in patients with recent onset T1DM and associated with a rapid decline in C-peptide, the most widely accepted measure of endogenous insulin production (14). SIGLEC-1^+^CD45^+^Lin^−^ MCs were suggested as an indicator for early diagnosis and monitoring of therapeutic efficacy in T1DM (15). CCR2^+^CD11b^+^Ly6G^low^Ly6C^high^ MCs were proposed for pathogenesis of early lesions of diabetic retinopathy (16). These observations support an important role for MC heterogeneity in diabetes-associated immune dysregulation. However, the immunological reprogramming of Ly6C-defined MC subsets in diabetes has not been systematically characterized.

Our recent findings (17, 18) by analyzing immune transcriptomes in flow cytometry-sorted Ly6C^high^ and Ly6C^low^ MC subsets from hyperlipidemia (HL, ApoE^−/−^) and hyperhomocysteinemia (HHcy) *Cbs^−/−^* mouse peripheral blood demonstrated that Ly6C^high^ MCs displayed enriched inflammatory pathways, favored to be differentiated into MΦ and lower Ag-presenting capacity. Ly6C^low^ MCs displayed anti-inflammatory/atherogenic features manifested activated T-cell signaling pathways and higher Ag-presenting capacity (17, 18). HHcy and *ApoE*^−/−^ confer upon both subsets with augmented pro-atherogenic/inflammatory function and Ag-presenting capacity (3, 4, 17–19). These findings suggest that systemic metabolic disorders can substantially reshape monocyte subset function. However, whether diabetes induces similar or distinct immunological reprogramming in Ly6C^high^ and Ly6C^low^ MC subsets remains unclear.

The primary goal of this study is to delineate the distinct immunological reprogramming of Ly6C MC subsets in diabetes. Specifically, we aim to examine how the diabetes modulates three key immune activation signals in innate and adaptive immune response, including signal 1 antigen (Ag)-presenting, signal 2 immune checkpoint, and signal 3 cytokine, and their upstream transcription factors (TF) to drive systemic inflammation. We engaged TF characterization for the identified target genes and modeled immunological transcriptional signaling in diabetes MCs.

## Materials and methods

**Figure 1:**
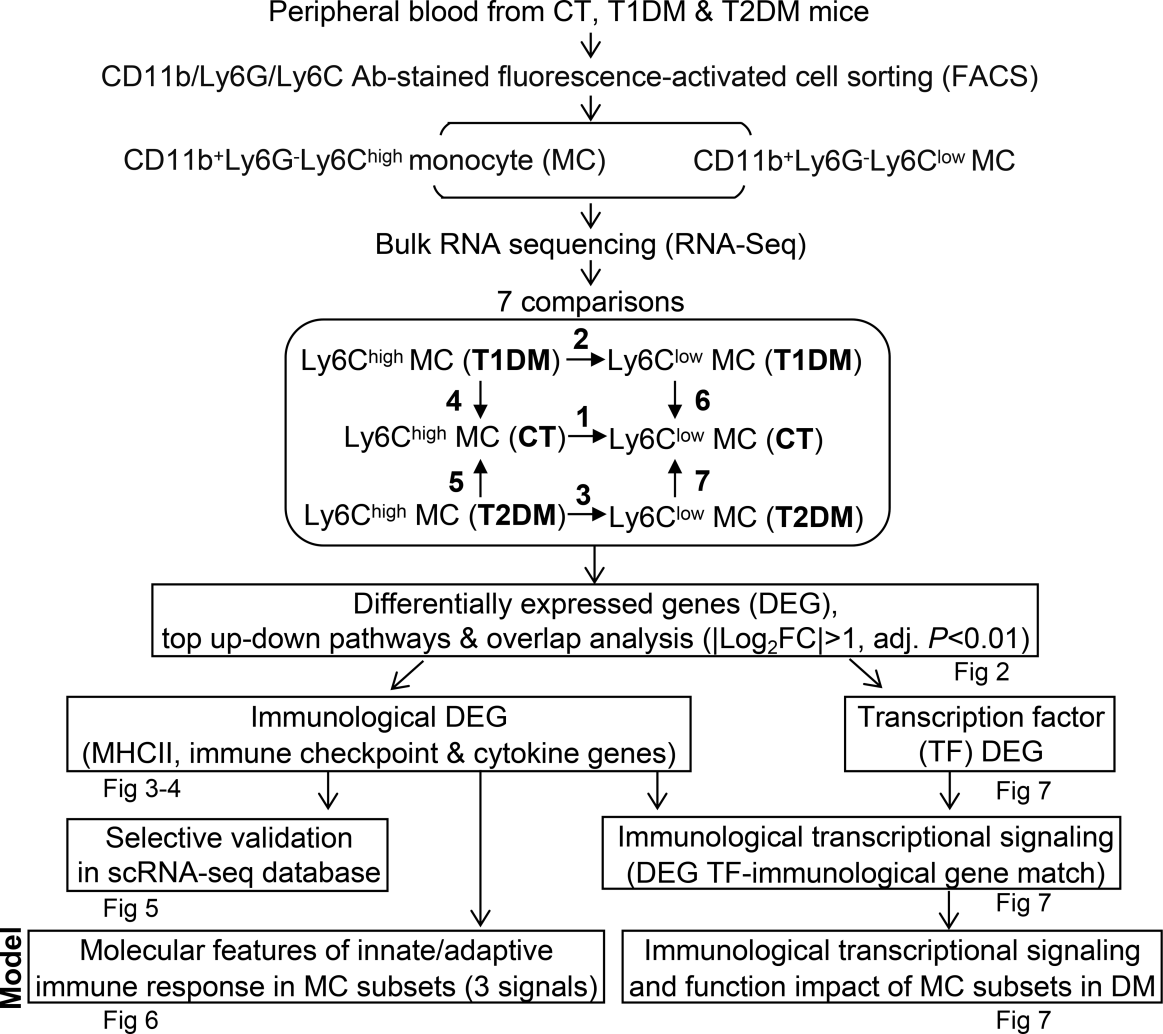
Overall strategy of identifying immunologic molecular targets and transcriptional signaling in the Ly6C MC subset from diabetic mice. RNA-Seq was performed in Ly6C^high^ (CD11b^+^Ly6G^−^Ly6C^high^) and Ly6C^low^ (CD11b^+^Ly6G^−^Ly6C^low^) MC isolated by flow cytometry sorting from peripheral blood of C57/BL6 control (CT), T1DM, and T2DM mice. Transcriptome data were analyzed by performing seven pairs of comparisons between the MC subset and mouse groups. DEG were identified by using the criteria of |Log2FC| more than 1 (2-FC) and adjusted P value less than 0.01. Top 10 canonical pathways were recognized by top-down analysis with |Z-score|>2, P value<0.05. Overlapped analysis were performed for canonical pathways between groups. Three sets of immunological DEG (MHCII, immune checkpoint, and cytokine genes) were identified and selected for validation in the mouse MC scRNA-seq database. TF DEG matched with immunological DEG were identified for the establishment of MC subset transcriptional signaling in DM. Models for molecular features of innate/adaptive immune response and immunological transcriptional signaling and function impact of MC subsets in DM were established.

We briefly summarize the overall strategy of identifying diabetic immunological genes and transcriptional signaling in the Ly6C MC subset from T1DM and T2DM mice in **Figure 1**. Bulk RNA-Seq was performed in Ly6C^high^ and Ly6C^low^ MCs isolated by flow cytometry sorting from peripheral blood of C57/BL6 control (CT) and diabetic mice. Transcriptome data were analyzed by performing seven pairs of comparisons between MC subsets and mouse groups. Immunologically significant differentially expressed genes (DEG) and canonical pathways were identified and followed with overlap analysis. Representative immunological DEG were validated with the mouse MC scRNA-seq database. Transcriptional factor (TF) DEG matched with immunological DEG were identified to establish the transcriptional signaling. Molecular features of DM MC subsets were established (details described below).

### Diabetic mouse model

C57/BL6 and diabetic mice (n=5) were fed a normal chow diet. C57/BL6 mice on rodent chow were used as CT, C57/BL6 mice treated with streptozotocin (STZ, intraperitoneally, 50 mg/kg per day for 5 successive days at the age of 8 weeks) as T1DM, and *db*/*db* mice treated with homozygous mutation of leptin receptor as T2DM. Animals were sacrificed at 20–22 weeks of age for blood collection after euthanization. All experiments were conducted in accordance with the National Institutes of Health Guidelines for the Care and Use of Laboratory Animals and with approval from Temple University School of Medicine Institutional Animal Care and Use Committee.

### Flow cytometry and cell sorting

**Figure 2:**
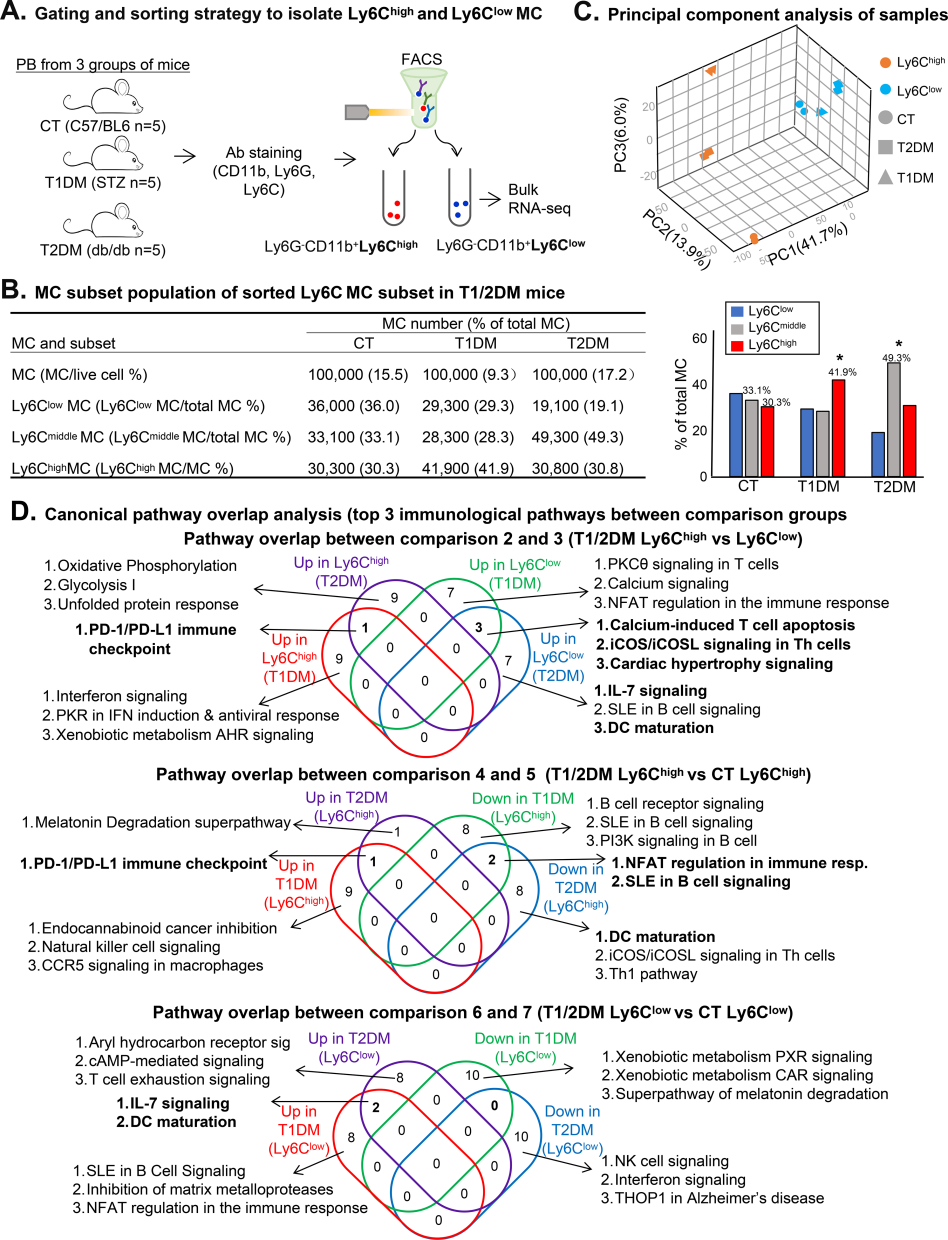
RNA-Seq analysis and DEG identification from blood Ly6C^high^ and Ly6C^low^ MCs of T1DM and T2DM mice. Male C57/BL6, T1DM (STZ ip 5 days), and T2DM (*db/db*) mice at age 22 weeks were used in this study (n=5). Bulk RNA-seq was performed on Ly6C MC subsets isolated by antibody staining and fluorescent-activated cell sorting (CD11b^+^Ly6G^−^Ly6C^high^ and CD11b^+^Ly6G^−^Ly6C^low^). **(A)** Gating and sorting strategy to isolate Ly6C^high^ and Ly6C^low^ MC. **(B)** MC subset population of the sorted Ly6C MC subset in T1/2DM mice. Mouse CD11b^+^Ly6G^−^ were classified as MCs (100,000 MCs per group) and sorted for the Ly6C MC subset by flow cytometry. **(C)** Principal component analysis of samples. PCA analysis incorporated 12 samples from 6 groups of MC subsets {Ly6C^high^(CT), Ly6C^low^(CT), Ly6C^high^(T1DM), Ly6C^low^(T1DM), Ly6C^high^(T2DM) and Ly6C^low^(T2DM)}. **(D)** Canonical pathway overlap analysis (top 3 immunological pathways between comparison groups).

MC subsets, Ly6C^high^ (CD11b^+^Ly6G^−^Ly6C^high^) and Ly6C^low^ (CD11b^+^Ly6G^−^Ly6C^low^), were isolated by flow cytometry sorting from peripheral blood cells pooled from five mice in each group (**Figure 2A**) as we previously described (5, 17, 18, 20). Bulk RNA-seq analysis was performed on 100,000 sorted cells for each subset. MC subset distribution was characterized (**Figure 2B**).

### RNA sequencing and data processing

RNA from purified MC subsets was extracted from individual mice and pooled (five mice each group) and subjected for bulk RNA sequencing in duplicate on NextSeq 500 for CT mice and the Illumina HiSeq 4000 sequencer for diabetic mice. The raw RNA-seq data contain 40 million reads in each sample and were analyzed by using the R Programming language. Raw sequencing reads in FASTQ format were processed using Kallisto (v0.45) for pseudoalignment and transcript quantification (17). Gene-level expression values were obtained using tximport (1.12.3) in R (3.6.1) to summarize transcript-level estimates to gene counts and transcript per kilobase per million mapped read (TPM) values for downstream analysis.

### Principal component analysis and comparisons

Intensive bioinformatic analysis was performed as described (17). We incorporated 12 samples from 6 groups of MC subsets, 2 datasets for each group [Ly6C^high^ (CT), Ly6C^low^ (CT), Ly6C^high^ (T1DM) and Ly6C^low^ (T1DM) Ly6C^high^ (T2DM) and Ly6C^low^ (T2DM)] for PCA.

Seven comparisons were performed to identify DM-altered DEG as briefly explained in **Figure 1**) CT Ly6C^high^ vs CT Ly6C^low^, 2) T1DM Ly6C^high^ vs T1DM Ly6C^low^, 3). T2DM Ly6C^high^ vs T2DM Ly6C^low^, 4. T1DM Ly6C^high^ vs CT Ly6C^high^, 5. T2DM Ly6C^high^ vs CT Ly6C^high^, 6. T1DM Ly6C^low^ vs CT Ly6C^low^, 7. T2DM Ly6C^low^ vs CT Ly6C^low^.

### Identification of DEG and canonical pathways

DEG were identified by using the Bioconductor suite of Limma packages in RStudio software with the criteria of |fold change (FC)|>2 and adjusted *P*-value<0.01. The heatmap was generated in RStudio using the “heatmap” package to present the expression levels of DEG. Ingenuity Pathway Analysis (IPA) version 7.1 was used to identify functional pathways and matched TF DEG with their corresponding immunological DEG. Venn diagrams were displayed to present the overlaps of pathways between comparisons.

### Immunological feature analysis

Firstly, we characterized transcriptome changes in the three classical signals of adaptive immune activation, including signal 1 antigen recognition, signal 2 immune checkpoint, and signal 3 cytokine stimulation (3, 8, 19). Heatmap for the identified immunological DEG was generated in RStudio using the “pheatmap” package with color density indicating the average expression level of a given gene normalized by z-score. Adaptive immunological features of the immunological DEG are justified by published literature.

### Validation of representative immunological DEG in the MC scRNA-seq dataset

We searched for public scRNA-seq datasets for mouse MC subset transcriptome information to validate mouse MC subset DEG identified in our study and selected the NO. E-MTAB-7678 dataset (21). The E-MTAB-7678 dataset used FACS-sorted mouse lung Ly6C^low^ and Ly6C^high^ MCs (CD45^+^F4/80^+^CD11c^−^Ly6C^low^CD64^−^ and CD45^+^F4/80^+^CD11c^−^Ly6C^high^CD64^−^ cells) and is available in the ArrayExpress database (https://www.ebi.ac.uk/gxa/sc/experiments/E-MTAB-7678/results/tsne).

CD45 and CD11c were used to marker leukocytes and myeloid cells/dendritic cells. F4/80 was used as mouse Mφ and MC markers to define myeloid lineage cells. CD64 (FcγRI) markers for activated Mφ to capture only the CD64^−^ MC. This dataset is comparable to ours because it focuses on well-defined MC subsets, abundant in the MC subset population with 443 Ly-6C^low^ MCs and 370 Ly6C^high^ MCs, and good in-depth sequencing information with a maximum of 3,000 detected genes (21).

### Identification of upstream TF for identified immunological DEG

TFs were identified by IPA software. Immunological DEG were matched with TF DEG by using IPA upstream analysis. The transcriptional regulatory relationship between TF DEG and immunological DEG was justified by IPA overlapped |z-score|>2 and P<0.01. Models of potential transcriptional axes in diabetic MC subsets describe potential transcriptional regulatory machinery.

## Results

### Ly6C^high^ MCs were elevated in T1DM mice, whereas Ly6C^middle^ MCs were increased in T2DM mice

We found that Ly6C^high^ MCs were increased from 30.3% in CT mice to 41.9% in T1DM, and that Ly6C^middle^ MCs were increased from 33.1% in CT mice to 49.3% in T2DM mice (**Figure 2B**).

In bulk RNA-Seq analysis, we obtained 40 million reads and 16,476 normalized genes in each group. PCA study demonstrated a distinct separation of the two MC subsets (**Figure 2C**). The PC1 axis separated Ly6C^high^ and Ly6C^low^ MCs in both mouse groups and explained 41.7% of the variance. However, the PC2 axis, which explains 13.9% of the variance, separated the Ly6C^high^ MCs from the DM and CT groups, but not the Ly6C^low^ MC.

T1DM induced 264-upregulated/500-downregulated DEG in Ly6C^high^ and 255-upregulated/356-downregulated in Ly6C^low^ MCs (4/6 comparison). T2DM induced 395-upregulated/982-downregulated DEG in Ly6C^high^ and 435-upregulated/400-downregulated in Ly6C^low^ (5/7 comparison). When comparing between the two subsets within the same disease model, we found that Ly6C^high^ MCs exhibited 1,512-upregulated/2,275-downregulated DEG in T1DM, and 1,612-upregulated/2,350-downregulated in T2DM mice (2/3 comparison) (**Supplementary Figure 1**).

### Ly6C^high^ MCs exhibited exacerbated inflammatory pathways in T1DM and suppressed adaptive immunity in both T1/T2DM

We listed the top 10 up/down significantly enriched canonical pathways by conducting top-down analysis of DEG identified from comparisons 2 to 7 from the IPA study (**Supplementary Figure 2**), as comparison 1 results were discussed in our previous publication (17, 18). **Figure 2D** summarizes pathway overlap analysis and further justified the following discovery.

We found that inflammatory pathways, interferon signaling, PKR in IFN induction and antiviral response, Toll-like receptor signaling, neuroinflammation signaling, and iNOS signaling were activated in T1DM Ly6C^high^ MCs compared to Ly6C^low^ MCs (**Supplementary Figure 2A**). Additionally, inflammatory pathways, NK cell signaling, CCR5 signaling in macrophages, and IL-15 production were further activated by T1DM vs CT Ly6C^high^ MCs (**Supplementary Figure 2C**). Two of the above activated pathways, IL-7 signaling and DC maturation, were overlapped in Ly6C^low^ MCs from T1/2DM mice (**Supplementary Figures 2E, F**; **Figure 2D**).

Another important discovery is that adaptive immune response pathways were enriched in Ly6C^low^ MCs from both T1/2DM mice. For example, calcium-induced T-cell apoptosis, PKCθ signaling in T cells, iCOS-iCOSL signaling in Th cells, NFAT in regulation of the immune response, and B-cell receptor signaling were upregulated in T1DM Ly6C^low^ MCs (**Supplementary Figure 2A**). Similarly, calcium-induced T-cell apoptosis and signalings for CCR5, IL-7, Th cell iCOS-iCOSL, and B-cell SLE were enriched in T2DM Ly6C^low^ MCs (**Supplementary Figure 2B**). Wherein, calcium-induced T-cell apoptosis and iCOS/iCOSL signaling in Th cells were both downregulated in Ly6C^high^ MCs from T1/2DM mice (**Supplementary Figures 2A, B**; **Figure 2D**). In the same direction, two adaptive immune pathways (NFAT regulation immune response and SLE in B-cell signaling) were suppressed in T1/2DM Ly6C^high^ MCs (**Supplementary Figures 2C, D**; **Figure 2D**). Furthermore, T1DM mice triggered the activation and maturation of DC, as well as signalings for IL-7, B-cell SLE, and NFAT in Ly6C^low^ MCs (**Supplementary Figure 2E**). Moreover, in Ly6C^low^ MC, T2DM induced DC maturation and signalings for T-cell exhaustion and IL-7 and chemokine activation (**Supplementary Figure 2F**). This pattern is consistent with the autoimmune and chronic inflammatory milieu of T1DM, in which sustained interferon-related and innate immune activation contributes to persistent immune-mediated tissue injury.

More importantly, PD-1/PD-L1, as a suppressive immune checkpoint molecule pair, was elevated in T1/2DM Ly6C^high^ MCs in comparisons both between and within MC subsets (**Supplementary Figures 2A–D**). This is also demonstrated by overlap analysis (**Figure 2D**).

Several metabolic pathway activities were enriched in both subsets by T1/2DM. For example, xenobiotic metabolism AHR/PXR and corticotropin-releasing hormone were upregulated in T1DM Ly6C^high^ vs Ly6C^low^ MCs (**Supplementary Figure 2A**). Oxidative phosphorylation, glycolysis I, guanosine nucleotide degradation III, gluconeogenesis I, fatty acid Î²-oxidation I, eicosanoid signaling, and triacylglycerol biosynthesis were activated in T1DM Ly6C^high^ compared to Ly6C^low^ MCs (**Supplementary Figure 2B**).

### MHC-II were reduced in Ly6C^high^ MCs and further suppressed by T2DM

**Figure 3:**
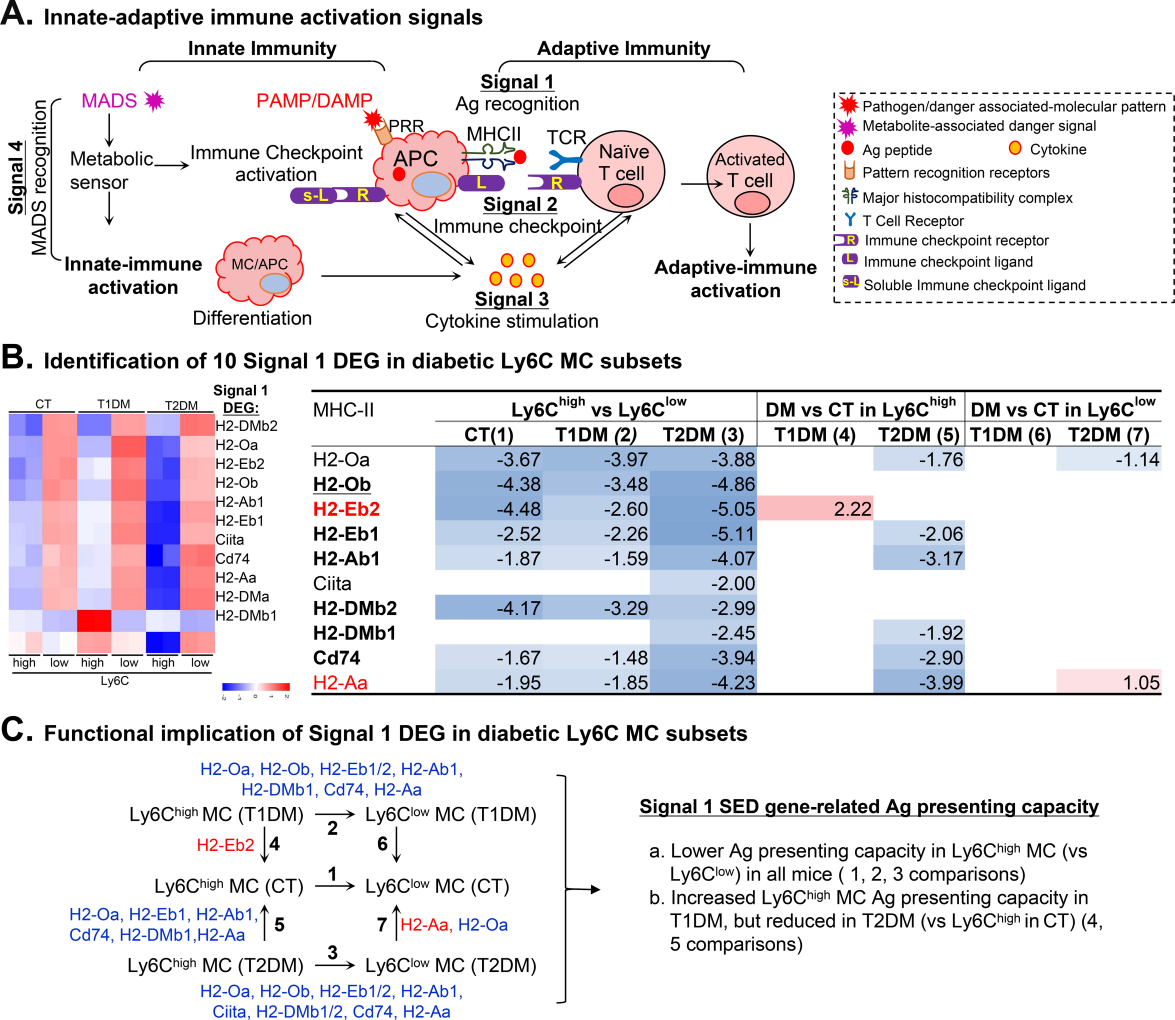
Identification and functional implication of immune activation signal 1 DEG in diabetic Ly6C MC subsets. **(A)** Innate-adaptive immune activation signals. Four immune activation signals are required for a fully achieved adaptive-immune activation. The initial immune response is stimulated by either the classical pathogen/danger associated-molecular pattern (PAMP/DAMP) via pattern recognition receptors (PRR) or the newly recognized metabolite-associated danger signal (MADS) via metabolic sensor (MS). In signal 1, MHC molecules present Ag peptide to TCR. The following signal 2 continued with the ligation of immune checkpoint ligand (L) and receptor (R). Signal 3 amplifies the immune response via cytokine secretion to achieve a full adaptive-immune activation. Immune checkpoint activation can result in innate-immune activation via the induction of soluble checkpoint ligand (s-L). **(B)** Identification of signal 1 DEG. Heatmap color density indicates the average expression level of a given gene normalized by z-score. The red and blue rainbow background in the table represents the gradient of increased/reduced fold change, as explained in the heatmap (log_2_FC>1 and log_2_FC<-1, respectively). **(C)** Functional implication of signal 1 DEG (n) Flowchart shows seven comparisons of MHC-II gene expression in MC subsets. The identified MHC-II DEG of each set of comparison are listed with the red and blue letters highlighting the up- and downregulated genes. MHC-II DEG-implicated functions are summarized.

Antigen presentation (signal 1) is a critical physiological bridge between innate and adaptive immunity. Our analysis revealed a widespread suppression of this capacity in inflammatory MC. Expression profiles of 10 DEG major histocompatibility complex class II (MHC-II) genes are identified through seven comparisons (**Figure 3A**). MHC-II genes H2-Oa, H2-Ob, H2-DMb2, H2-Eb1/2, H2-Ab1, H2-Aa, and Cd74 exhibited a reduced expression in Ly6C^high^ MCs compared with Ly6C^low^ MCs in all mice, with exception of 2 MHC-II genes (Ciita and H2-DMb1) which were reduced only in T2DM mice. Crucially, the diabetic microenvironment further exacerbated this defect. While H2-Eb2 was elevated in T1DM Ly6C^high^ MCs, in T2DM mice specifically, the expression of core MHC-II machinery was profoundly suppressed, with H2-Oa, H2-Eb1, H2-Ab1, H2-DMb1, Cd74, and H2-Aa showing further reductions. This transcriptional silencing indicates a physiological shift where diabetic Ly6Chigh MCs lose their ability to efficiently present antigens, potentially contributing to the immune paresis and delayed tissue repair commonly observed in chronic diabetic complications (**Figure 3B**).

“When directly comparing the transcriptomic landscape between the two diabetic milieus, we observed a striking divergence in antigen-presenting capacities. While T1DM Ly6C^high^ MCs maintained a relatively stable signal 1 profile, the T2DM environment exerted a profound and specific suppressive effect on these cells, uniquely downregulating the PAX5-CIITA regulatory axis and MHC-II expression. This head-to-head comparison reveals that while both conditions disrupt monocyte homeostasis, T2DM drives a more pronounced state of immune paresis regarding antigen recognition.”

### Immune checkpoint receptors were reduced in Ly6C^high^ MCs and further suppressed in both T1/2DM

**Figure 4:**
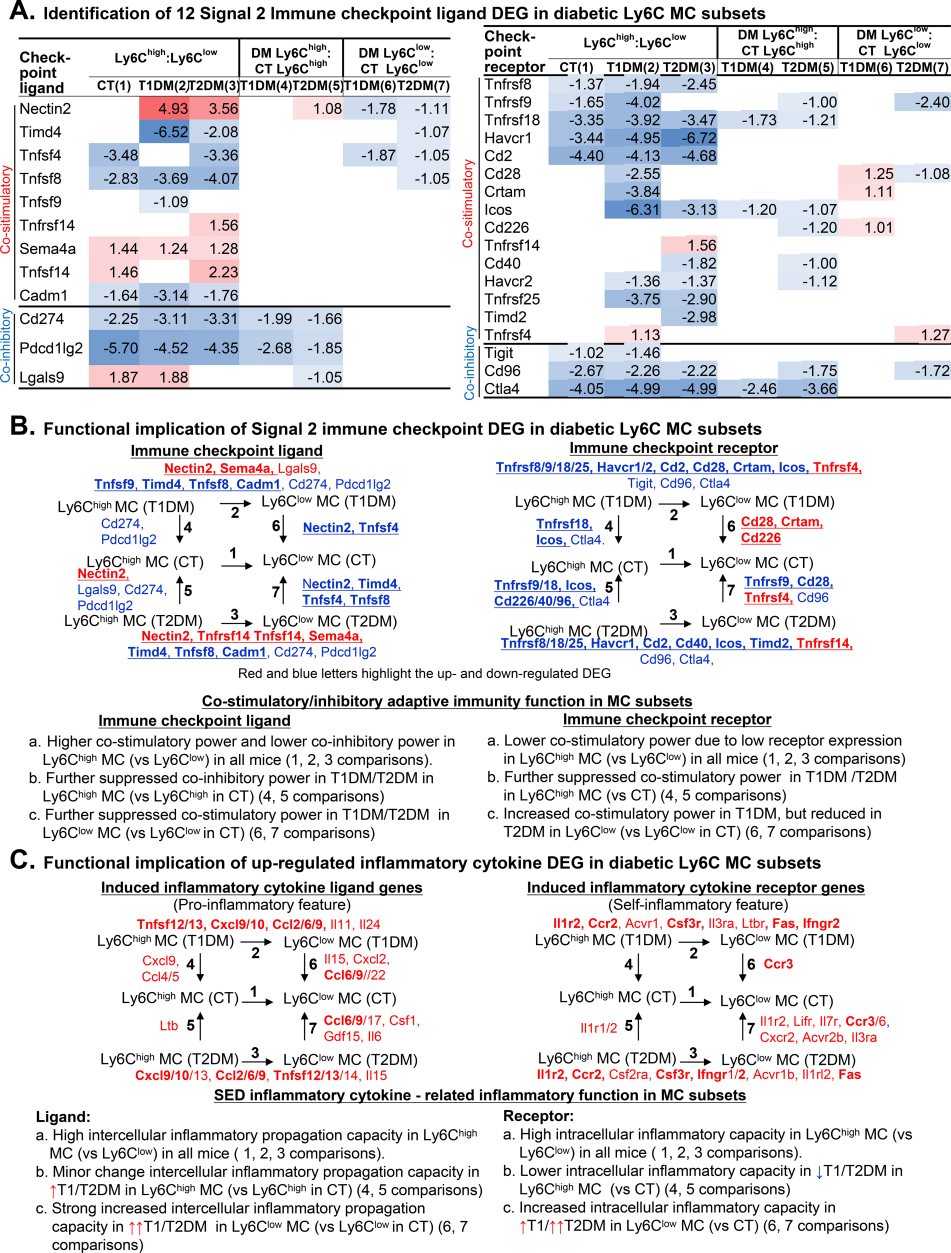
Identification of signal 2 immune checkpoint DEG and functional implication in diabetic Ly6C MC subsets. **(A)** Identification of signal 2 DEG immune checkpoint ligand and receptor genes. The red/blue rainbow background in the table represents the gradient of increased/reduced fold change (log_2_FC>1 and log_2_FC<-1, respectively). **(B)** Functional implication of signal 2 immune checkpoint DEG. Flowchart shows seven comparisons of immune checkpoint gene expression in MC subsets. Immune checkpoint DEG implicated functions are summarized. Bolded underlines highlight the co-stimulatory immune checkpoint. **(C)** Functional implication of signal 3 cytokine DEG. Red and blue letters highlight the representative up- and downregulated genes. Bolded letters display pro-inflammatory cytokine.

To understand how diabetes alters MC-mediated immune signaling (signal 2), we analyzed 30 significantly differentially expressed immune checkpoint genes (18 receptors and 12 ligands) (**Figure 4A**). Rather than isolated gene changes, we observed a systematic uncoupling of co-stimulatory networks. The most obvious change was a broad reduction in co-stimulatory receptors. Ly6C^high^ MCs consistently downregulated critical co-stimulatory receptors (such as *Icos*, *Havcr2*, and *Tnfrsf25*) in both T1DM and T2DM mice compared to healthy controls. Physiologically, this broad down-regulation suggests that diabetic Ly6C^high^ MCs exist in a state of altered immune reactivity—capable of circulating and infiltrating tissues, but impaired in their ability to engage in balanced, reciprocal communication with T cells.

Regarding checkpoint ligands, Ly6C^high^ MCs displayed a distinct functional dichotomy. While they expressed higher levels of the stimulatory ligand *Sema4a*, they exhibited significantly lower levels of co-inhibitory ligands, particularly *Cd274* (PD-L1) and *Pdcd1lg2* (PD-L2), a defect further exacerbated by T1/2DM. This specific profile, high stimulatory potential (via *Sema4a*/*Nectin2*) coupled with reduced co-inhibitory “brakes” (*Cd274*), suggests that while diabetic Ly6C^high^ MCs are less responsive to external regulation (due to low receptors), they retain a “loud” stimulatory phenotype that may drive unchecked immune activation in other cells (**Figure 4B**).

We observed reduced immune checkpoint receptor genes in Ly6C^high^ MCs in all mice, which was further suppressed in T1/2DM. These data led us to hypothesize a lower immune reactivity of differentiation potential for Ly6C^high^ MCs and in diabetes.

### Differential inflammatory cytokine-related property of MC subsets and changes in T1/2DM

We found that pro-inflammatory (intercellular inflammatory propagation capacity) cytokine Tnfsf12/13, Cxcl9/10, and Ccl2/6/9 were highly expressed and elevated by more than 2-fold in Ly6C^high^ MCs compared with Ly6C^low^ MCs in all mice. Moreover, intercellular inflammatory propagation capacity cytokine Il11 was elevated in Ly6C^high^ MCs compared with Ly6C^low^ MCs in CT and T1DM. Intercellular inflammatory propagation capacity cytokine Il15 and Tnfsf14 were increased in Ly6C^high^ MCs compared with Ly6C^low^ MCs in CT and T2DM mice. These cytokines are mostly relevant for determining the pro-inflammatory feature of Ly6C^high^ MCs and in DM conditions (**Figure 4C**; **Supplementary Figure 3**).

However, we also observed favorable anti-inflammatory changes for cytokines in Ly6C^high^ MC. For example, pro-inflammatory cytokine ligands (Il6, Cxcl12, Ccl4/5/22, Tnfsf8, and Ifng) were reduced in Ly6C^high^ MCs compared with Ly6C^low^ MCs in all mice.

The influence of T/2DM to cytokine expression in MC subsets is complex. T1/2DM increased Ccl6/9 in Ly6C^low^ MC but decreased the expression of pro-inflammatory cytokines Gdf3, Cxcl13, and Ccl22 in Ly6C^high^ MC, and Tnfsf4 and Cxcl10 in Ly6C^low^ MC. Otherwise, T2DM-induced pro-inflammatory cytokine Ccl17, Csf1, Gdf15, and Il6 were elevated in Ly6C^low^ MCs compared with CT Ly6C^low^ MC.

Consistently, we found 6 induced inflammatory cytokine receptor genes (Il1r2, Ccr2, Il13ra1, Csf3r, Ifngr2, and Fas) and 10 reduced cytokine receptor genes (Ccr6, Il9r, Il21r, Lepr, Il5ra, Il18rap, Il27ra, Il18r1, Il12rb2, and Il2rb) in Ly6C^high^ MCs compared to Ly6C^low^ MCs in all mice. In addition, Il1r1/2 were elevated in T2DM Ly6C^high^ MC, Ccr3 was elevated in T1DM Ly6C^low^ MC, and Il1r2, Lifr, Il7r, Acvr2b, Il3ra, Ccr3/6, and Cxcr2 were elevated in T2DM Ly6C^low^ MC.

Ly6C^high^ MCs display reduced receptor expression and innate immune responses compared to Ly6C^low^ MCs in all mice (one, two, and three comparisons). In both T1DM and T2DM conditions, Ly6C^high^ MCs exhibit reduced receptor expression compared to CT mice (four and five comparisons). In T1DM and T2DM, Ly6C^low^ MCs also show decreased receptor expression when compared to CT mice (six and seven comparisons).

### Cross-platform scRNA-seq analysis validates key immunological transcriptional signatures

**Figure 5:**
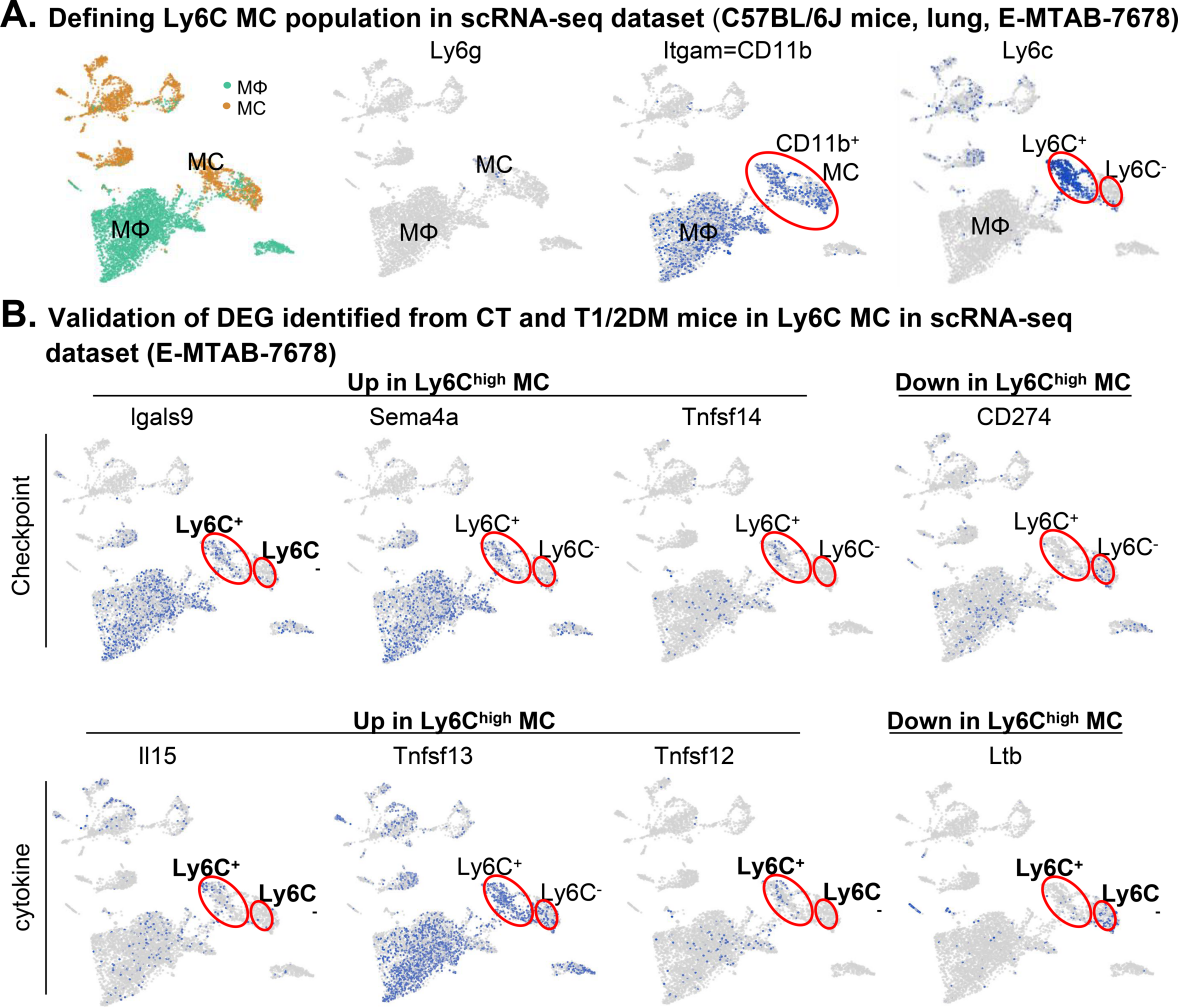
Validation of selected MC subset immunological DEG in the scRNA-seq database. **(A)** Defining the Ly6C MC population in the scRNA-seq dataset (C57BL/6J mice, lung). Single-cell RNA-seq (ScRNA-seq) data of CD64^+^ mononuclear cells in lungs of C57BL/6J mice were obtained (NO. E-MTAB-7678). Ly6C^+^ MC(Ly6G^-^CD11b^+^Ly6C^+^) and Ly6C^−^ MCs (Ly6G^-^CD11b^+^Ly6C^−^) were distinguished according to their deferential expression of Ly6c. **(B)** Validation of DEG identified in Ly6C MC subsets in our CT mice. UMAP displays the deferential expression of selected MC subset immunological DEG in C57BL/6J mice identified in our study.

We employed a scRNA-seq dataset derived from the lungs of C57BL/6J mice from EMBL-EBI under accession number E-MTAB-7678 (PMID: 31481690) to validate representative immunological DEG identified in this study. As shown in **Figure 5**, consistent with our RNA-Seq data analysis, immune checkpoint DEG Lgals9, Sema4a, and Tnfsf14 were confirmed for upregulated expression and Cd274 for downregulated expression in Ly6C^+^ MCs, which was defined as Ly6G^−^CD11b^+^Ly6C^+^ MC. Similarly, cytokine DEG Il15, Tnfsf13, and Tnfsf12 displayed a higher level expression, and Ltb was lower in Ly6C^+^ MC. This validation process ensures the reliability and reproducibility of our findings regarding the distinct transcriptional signatures in MC subpopulations.

### Model of T1/2DM-impacted innate/adaptive immune response in mouse Ly6C MC subsets

**Figure 6:**
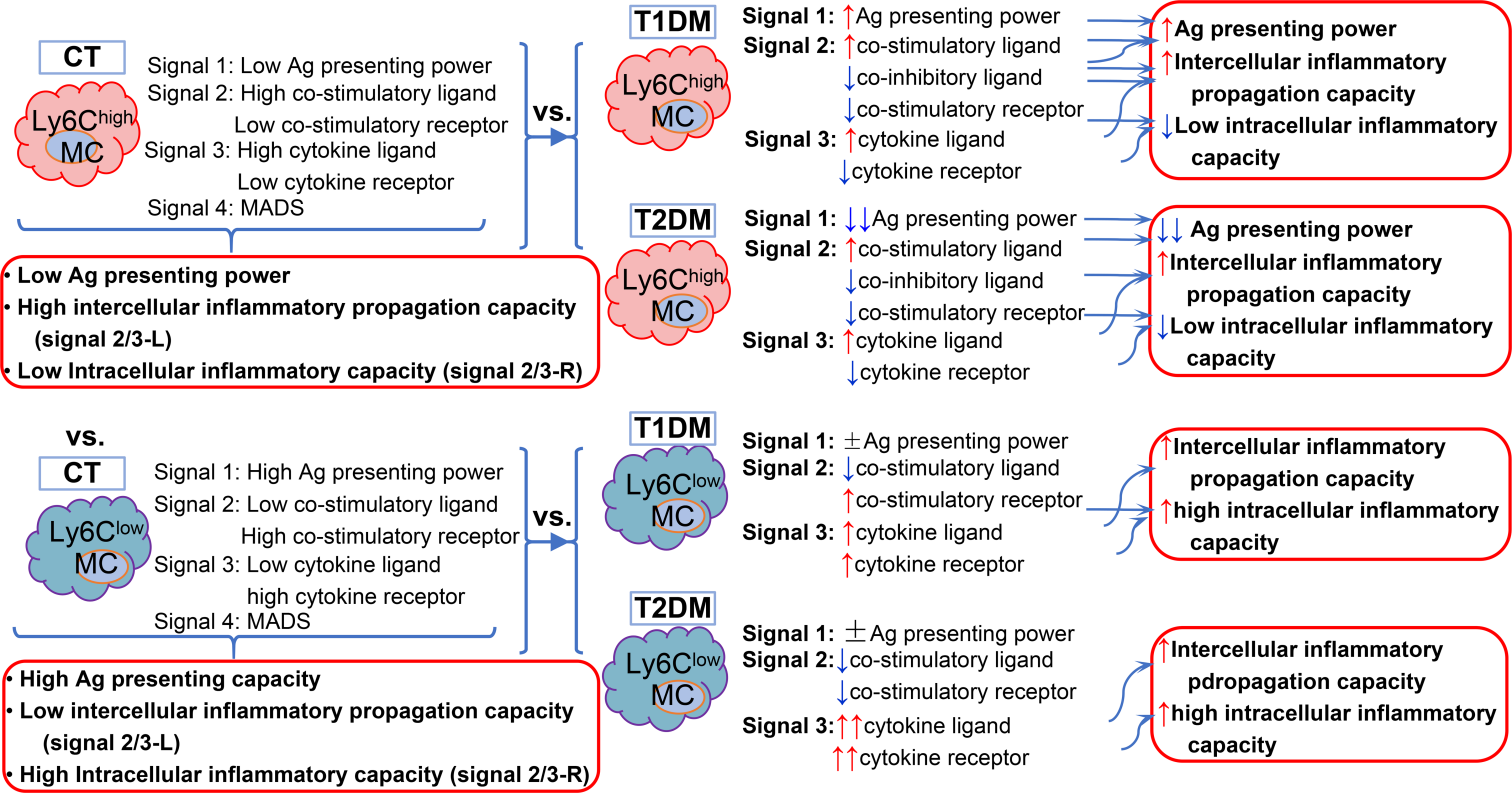
Immunology feature of mouse Ly6C MC subsets and T1/2DM-impacted response. The model describes potential innate/adaptive immune response and functional changes in the T1/2DM condition. Arrows (red and blue) indicate the direction of immunology function changes (activated and suppressed).

We established a model to describe immunological feature and T1/2DM-impacted innate/adaptive immune response in mouse Ly6C MC subsets in **Figure 6** based on transcriptome analysis.

We conclude that Ly6C^high^ MCs have low Ag-presenting capacity, strong intercellular inflammatory propagation capacity, and low intracellular inflammatory capacity, while Ly6C^low^ MCs have high Ag-presenting capacity, anti-inflammatory capacity, and high intracellular inflammatory capacity.

In the T1DM condition, Ly6C^high^ MCs acquired a higher Ag-presenting capacity which contributes to increased adaptive immune response/reactivity. It had reduced co-inhibitory ligand and co-stimulatory receptor power and increased pro-inflammatory cytokine level, which strengthen the intercellular inflammatory propagation capacity, and decreased intracellular inflammatory capacity cytokine receptor power. On the other hand, T1DM Ly6C^low^ MCs displayed reduced co-stimulatory ligand power, enhanced co-stimulatory receptor power, and strengthened intercellular and intracellular inflammatory capacity, but no significant change in Ag-presenting capacity.

In the T2DM condition, Ly6C^high^ MCs decreased the co-inhibitory ligand and increased pro-inflammatory cytokine ligand power, which enhanced the intercellular inflammatory propagation capacity but reduced the co-stimulatory receptor power and intracellular inflammatory capacity cytokine receptor power related to further lower intracellular inflammatory capacity, and changed to a lower Ag-presenting capacity. Conversely, T2DM Ly6C^low^ MCs weakened the co-stimulatory power of both receptor and ligand and enhanced in intercellular and intracellular inflammatory capacity, but there was no significant change in Ag-presenting capacity.

Interestingly, signal 4, metabolic-associated danger signal (MADS), changes the immunological feature via altering signal 2/3 molecule expression independent from pattern recognition DAMP/PAMP recognition.

### Identification of 17 potential transcriptional axes modulating the immunological feature in diabetic Ly6C MC subsets

**Figure 7:**
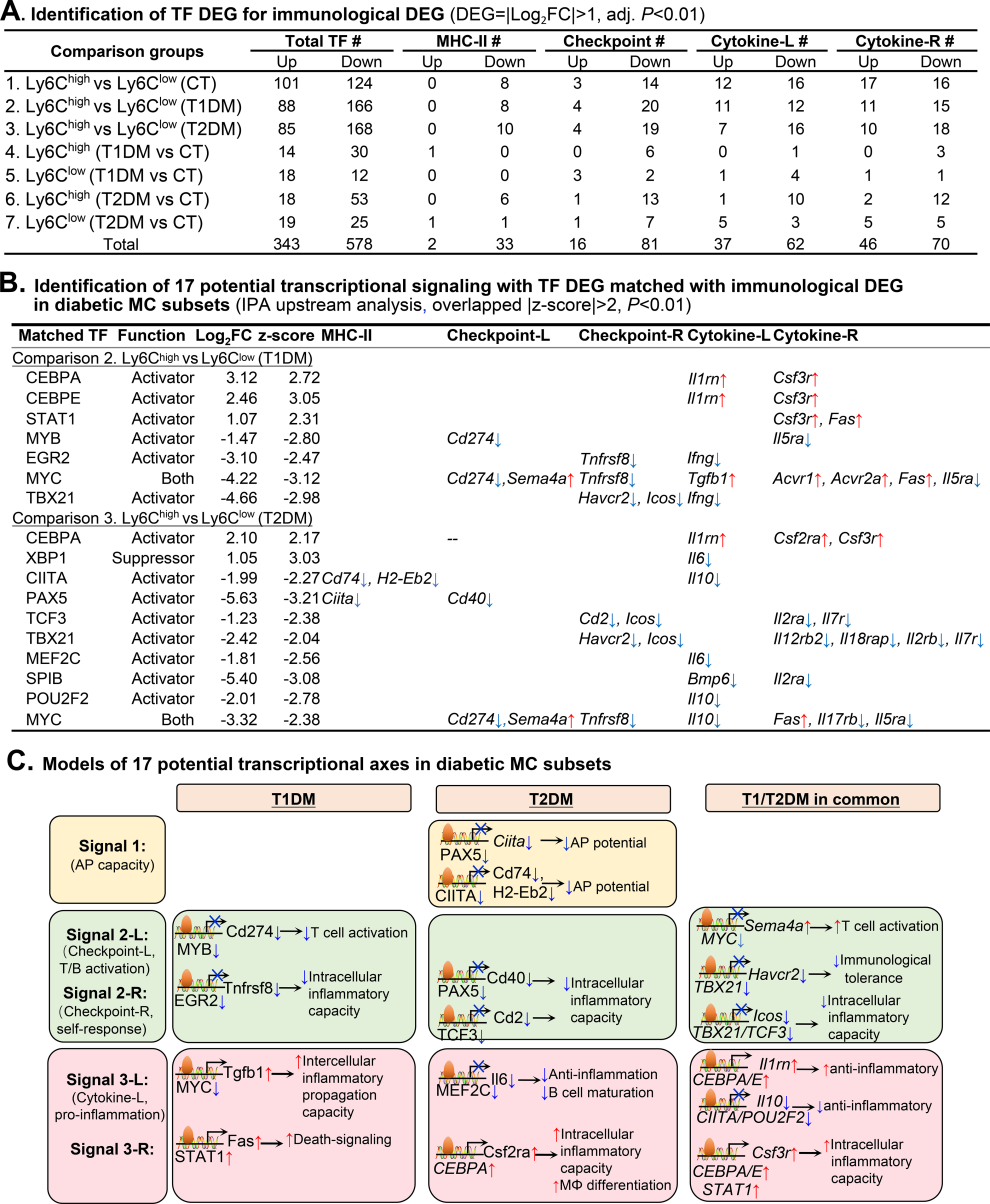
Identification of TF DEG for immunological DEG. **(A)** Identification of TF DEG for immunological DEG. Total 921 TF DEG were identified in seven comparison groups using the criteria of |Log_2_FC|>1 (2-FC) and adjusted P<0.01. **(B)** Identification of 17 potential transcriptional signaling with TF DEG matched with immunological DEG in diabetic MC subsets. Immunological DEG were matched with TF DEG by IPA upstream analysis. The transcriptional regulatory relationship between TF DEG and immunological DEG was justified by IPA overlapped |z-score|>2 and P<0.01. **(C)**. Models of 17 potential transcriptional axes in diabetic MC subsets. The model describes potential transcriptional regulatory machinery. Red up arrows indicate induced gene expression or immunological function. Blue down arrows indicate the suppression of its.

In the efforts to discover the transcriptional mechanism determining immunological feature in the Ly6C MC subset, we searched for differentially expressed TF in diabetic Ly6C MC subsets and identified totally 343-upregulated and 578-downregulated TF, which target 2-upregulated/33-downregulated MHC II, 16-upregulated/81-downregulated checkpoint, 37-upregulated/62-downregulated cytokine ligand, and 46-upregulated/70-downregulated cytokine receptor DEG from the seven comparisons (details in **Figure 7A**).

By matching the expressional change of immunological DEG with the function (transactivating or suppressing) of their corresponding upstream DEG TF, we identified 17 potential transcriptional signalings, which involve 12 trans-activating TF, 1 trans-suppressing TF (XBP1), and 1 dual-functional TF (MYC) (**Figure 7B**). In T1DM Ly6C^high^ MCs (comparison 2), increased trans-activating TF CEBPA, CEBPE, and STAT1 were associated with the upregulation of cytokine Il1rn, Csf3r, and Fas upregulation; reduced trans-activating TF MYB, EGR2, and TBX21 were associated with the downregulation of seven immunological genes; and reduced dual functional TF MYC was associated with Cd274, Tnfrsf8, and Il5ra suppression and Sema4a, Tgfb1, Acvr1, Acvr2a, and Fas induction. In T2DM Ly6C^high^ MCs (comparison 3), upregulated trans-activating TF (CEBPA) was associated with the upregulation of three cytokines (Il1rn, Csf2ra, and Csf3r), downregulated trans-activating TF (CIITA, PAX5, TCF3, TBX21, MEF2C, SPIB, and POU2F2) corresponded to 15 downregulated immunological genes, and TF MYC downregulation was associated with the downregulation of Cd274/Tnfrsf8/Il10/Il17rb/Il5ra and the upregulation of Sema4a/Fas. We also found that upregulated transcription repressor XBP1 was associated with the downregulation of Il6. The detailed list of DEG TF matching with the corresponding DEG is presented in **Supplementary Table 1**.

Based on above data, we established models to describe potential transcriptional axes underlying immunological feature change in diabetic MC subsets (**Figure 7C**). For example, the transcriptional axis of ↓PAX5-↓CIITA-↓Cd74/H2-Eb2 may weaken Ag-presenting capacity in T2DM Ly6C^high^ compared with T2DM Ly6C^low^. Axes ↓MYB-↓Cd274 in T1DM and ↓MYC-↑Sema4a in both T1/2DM may enhance the T-cell activation potential in Ly6C^high^ MC. In addition, axes of inhibited EGR2-Tnfrsf8, PAX5-Cd40, TCF3-Cd2, TBX21-Havcr2, and TBX21/TCF3-Icos may be responsible for suppressive immune checkpoint, further weakening adaptive immunity in both T1/2DM in Ly6C^high^ MCs compared with Ly6C^low^ MC.

Regulatory mechanisms underlying differential cytokine expression (signal 3) may involve axes of inhibited ↓MYC-↑Tgfb, ↓MEF2C↓Il6, and ↓CIITA/POU2F2-↓Il10 and increased ↑CEBPA-↑Csf2ra and ↑CEBPA/CEBPE/STAT1-↑Csf3r, which lead to increased intercellular inflammatory propagation capacity in both Ly6C^high^ and Ly6C^low^ MC, respectively.

When directly comparing the two diabetic settings, we observed a clear divergence in Ly6C^high^ MC adaptation. T1DM largely preserved, or only modestly altered, the signal 1 antigen-presenting program, consistent with an immune environment dominated by persistent inflammatory activation. In contrast, T2DM imposed a much stronger suppressive effect on the PAX5–CIITA–MHC-II axis, driving a state of antigen-presentation insufficiency. Thus, although both forms of diabetes disrupt monocyte homeostasis, T1DM is biased toward inflammatory activation, whereas T2DM is more strongly associated with impaired antigen-presenting function.

## Discussion

Inflammatory MC subset polarization is a hallmark of systemic and tissue inflammatory in T1DM and T2DM. We previously reported that the inflammatory Ly6C^high^ MC subset exacerbated diabetic vascular complications and accelerated atherosclerosis in T1/2DM mice (5). This study profiled transcriptomes of three classical immunological signals in MC subsets (Ly6C^high^ and Ly6C^low^) in CT, T1DM, and T2DM mice and established the underlying transcriptional signaling. We have six major findings: 1) Ly6C^high^ MCs displayed high intercellular inflammatory propagation capacity and low intracellular inflammatory capacity which were both further polarized by T1DM and T2DM. 2) Ly6C^low^ MCs displayed low intercellular inflammatory propagation capacity and high intracellular inflammatory capacity, and both acquired additional inflammatory potential in T1DM and T2DM. 3) Ly6C^high^ MCs presented lower Ag-presenting capacity in control mice, which was enhanced in T1DM but suppressed in T2DM. 4) Ly6C^high^ MCs were increased in T1DM mice, but Ly6C^middle^ MCs were increased in T2DM. 5) A total of 17 T1/2DM modulated transcriptional axes were modeled. These findings provide a comprehensive understanding of transcriptional regulation underlying immunological reprogramming in diabetic MC subsets and recommended potential therapeutic targets.

### Ly6C^high^ MCs exhibited high intercellular inflammatory propagation, but low intracellular inflammatory capacity compared with Ly6C^low^ MCs; both features were elevated in both subsets in T1/T2DM

We defined MC intercellular inflammatory propagation capacity based on immune checkpoint and cytokine ligand expression because immune checkpoint ligands deliver co-stimulatory signals that potentiate effector immune cell activation and that cytokine ligands directly mediate intercellular communication to amplify inflammatory responses.

Standard definitions of “pro-inflammatory” MCs often conflate cell-autonomous signaling states with outward effector functions. To precisely deconstruct the transcriptomic shifts in our diabetic models, we propose a bipartite conceptual framework. Intracellular inflammatory capacity is defined based on the expression of immune checkpoint and cytokine receptor expression, which refers to a cell’s ability to initiate and sustain inflammatory signaling within itself through immune checkpoint and cytokine receptors, encompassing processes such as transcriptional activation, NF-κB signaling, and cytokine production programs. In contrast, intercellular inflammatory propagation capacity refers to a cell’s ability to transmit or amplify inflammatory signals to other cells via immune checkpoint and cytokine ligands, which may be displayed on the cell surface or secreted, thereby influencing neighboring cells through mechanisms such as cytokine secretion, antigen presentation, and checkpoint modulation. Applying this framework, our data reveal that the diabetic milieu fundamentally uncouples these two dimensions: it profoundly impairs MC’s intercellular propagation (e.g., suppressed MHC-II and co-stimulatory ligands) while preserving or even exacerbating their intracellular inflammatory tone.

We found that Ly6C^high^ MCs in CT mice displayed high intercellular inflammatory propagation capacity based on higher levels of cytokine ligand expression (Il6, Cxcl12, Ccl4/5/22, Tnfsf8, and Ifng) and suppressed intracellular inflammatory capacity feature because of lower levels of co-stimulatory molecule receptor (Tnfrsf8/18, Havcr1, and Cd2) and cytokine receptor expression (Ccr6, Il9r, Il21r, Lepr, Il5ra, Il18rap, Il27ra, Il18r1, Il12rb2, and Il2rb) (**Figure 4**). In T1/2DM mice, Ly6C^high^ MCs enhanced intercellular inflammatory propagation capacity by downregulating co-inhibitory ligands Cd274 and Pdcd1lg2 and reduced the intracellular inflammatory capacity by decreasing the co-stimulatory receptor (Icos, Havcr2, and Tnfrsf25) and inflammation cytokine receptor (Il2rb, Il9r, and Ccr6) power. These data led us to conclude that the reduced co-stimulatory/cytokine receptors contribute to lower intracellular inflammatory capacity in Ly6C^high^ MCs and impact on a prolonged survival of Ly6C^high^ MCs in T1/2DM, leading to a more profound systemic and intercellular inflammatory propagation capacity status in T1/2DM.

The immune pathway analysis results underscore the role of diabetic Ly6C^high^ MCs in inflammatory response. We found that in control Ly6C^high^ MC, inflammatory canonical pathways, such as IFN, Toll-like receptor, and neuroinflammation signaling, are activated and that in T1DM Ly6C^high^ MC, NK cell and CCR5 signaling is further exacerbated, which aligns with their role in T1DM autoimmune-driven β-cell destruction. This heightened inflammatory feature of T1DM Ly6C^high^ MC may promote tissue damage through the release of cytokines such as IL-1β and TNF-α, as observed in T1DM (22). Our finding supported the involvement of IFN signaling in Ly6C^high^ MC-derived high intercellular inflammatory propagation capacity in both control and T1DM conditions. Our findings support the involvement of IFN-related signaling in shaping the heightened inflammatory phenotype of Ly6Chigh MC, particularly in T1DM, and place these cells within the broader autoimmune pathophysiology that drives chronic tissue injury and diabetic complications.

In addition, our pathway analysis data highlighted co-inhibitory PD-1/PD-L1 checkpoint pathway activation in Ly6C^high^ MCs compared with Ly6C^low^ MCs in the same mice in all groups. Moreover, the PD-1/PD-L1 pathway was further elevated by T1/2DM in Ly6C^high^ MCs compared with Ly6C^high^ MCs in control mice. This is consistent with our above discussion for reduced co-stimulatory/cytokine receptor expression in T1/2DM. Together, all contribute to lower intracellular inflammatory capacity in T1/2DM Ly6C^high^ MCs via a prolonged survival leading to a more profound systemic and pro-inflammatory response in T1/2DM. This finding suggests a benefit of MCs targeted anti-PD-L1 treatment for diabetic inflammation via suppressing PD-1 signaling and MC survival, which is consistent with cancer therapy, where PD-1/PD-L1 blockade enhances antitumor TC immunity (23). Interestingly, anti-PD-1 therapy was found beneficial and suppressed plaque-associated pro-inflammatory T cells via FcγR-mediated capture (e.g., Nivolumab) (24). On the other hand, negative reports suggested that anti-PD-L1 may exacerbate diabetes progression via IFN-γ^+^CD8^+^ T-cell infiltration and activating β-cell-killing (25, 26). Our findings emphasize the exacerbated activation of the PD-1/PD-L1 pathway in T1/2DM Ly6C^high^ MC, and its role in lower intrinsic intracellular inflammatory capacity and prolonged survival, suggesting that MC-targeted anti-PD-1/PD-L1 therapy may restrict inflammatory MC expansion and relevant inflammation in diabetes.

### Ly6C^low^ MCs displayed low pro-inflammatory and high intracellular inflammatory capacity features and acquired additional inflammatory potential in T1/2DM

Ly6C^low^ MCs are traditionally associated with anti-inflammatory or immunoregulatory functions under steady-state conditions. Our findings reveal that Ly6C^low^ MCs exhibit low intercellular inflammatory propagation capacity and high intracellular inflammatory capacity at baseline, as checkpoint/cytokine ligands (Tnfsf8, Ccl4/5/22, Il6, and Ifng) were reduced and checkpoint/cytokine receptors (Tnfrsf8/18, Havcr1, Cd2/Ccr6, Il9r, Il21r, Lepr, Il5ra, Il18rap, Il27ra, Il18r1, Il12rb2, and Il2rb) were induced in Ly6C^low^ MCs (**Figure 4**) compared with Ly6C^high^ MCs in the CT mice. The Ly6C^low^ MCs acquired intercellular inflammatory propagation capacity by upregulating co-stimulatory/cytokine ligand (Ccl6/9) and enhanced intracellular inflammatory capacity by upregulating co-stimulatory/cytokine receptors (Ccr3) in T1/2DM. This reflects a diabetes-disrupted immune homeostasis in MC subsets. Immunological reprogramming in both Ly6C^high^ and Ly6C^low^ MCs highlights their role in contributing to diabetes-associated systemic and tissue inflammatory response (5, 27).

The T1/2DM microenvironment exacerbates Ly6C^low^ MC intercellular inflammatory propagation capacity through upregulated cytokine ligand (Ccl6/9) and IL-7 signaling and DC maturation pathway, changing their native anti-inflammatory feature. IL-7 has been shown to enhance antigen presentation capacity in MC-derived DC and facilitated T-cell activation (28). Therefore, T1/2DM-activated IL-7 signaling and induced cytokine ligand expression in Ly6C^low^ MCs may contribute to autoimmune diabetes (T1DM) and metaflammation in T2DM via T-cell activation. Our findings suggest that the CCL6/9-CCR3 axis and IL-7 signaling represent the most promising near-term targets due to their direct roles in converting Ly6C^low^ MCs from anti-inflammatory to pro-inflammatory effector in diabetes.

### Lower Ag-presenting capacity in Ly6C^high^ MCs and further suppressed by T2DM

We observed that eight MHC-II genes (H2-Oa, H2-Ob, H2-DMb2, H2-Eb1/2, H2-Ab1, H2-Aa, and Cd74) were reduced in Ly6C^high^ MCs in all mice by comparing with Ly6C^low^ MC and that six MHC-II gene (H2-Oa, H2-Eb1, H2-Ab1, H2-Aa, H2-DMb1, and Cd74) were further reduced by T2DM. We concluded that Ly6C^high^ MCs have a lower Ag-presenting capacity, which was further suppressed by T2DM (**Figure 3B**). Similarly, we have previously reported a lower Ag-presenting capacity of Ly6C^high^ MCs in control mice which was also further suppressed in HHcy and HL conditions (17, 18). These findings are consistent with the observation showing reduced expression of HLA-DR/CD74 as well as homologs of mouse H2-Eb1/H2-Ab1/Cd74 MHC-II molecules, in all MC subpopulations in human T2DM (29). Reduced HLA-DR expression in inflammatory MCs was associated with the risk of severity and susceptibility to diabetes, cancer, or sepsis (30, 31). Furthermore, mice deficient in MHC-II-restricted antigen presentation by RORγt^+^ cells (MHC-II^+^RORγt^+^) developed severe intestinal inflammation potentially due to impaired regulatory T-cell (Tregs) polarization and Treg-dependent tolerance to gut microbiota (32, 33). Supportively, we and others have reported that HHcy and hyperlipidemia MCs suppressed Ag-presenting capacity, due to MHC-II reduction, and were connected to pro-inflammatory responses over tolerance and increased tissue damage risk (17, 18, 34, 35). Furthermore, MHC-II expression on professional innate immune cells (DC, MC, and Mϕ) and non-immune cells (endothelial cells and fibroblasts) (36, 37) enables CD4^+^ T cell-mediated inflammatory signaling (IFN-γ and CD40L) and perhaps confers “trained immunity” and functional reprogramming to elicit changed responses to subsequent inflammatory challenges. Interestingly, this T2DM-specific MHC-II suppression is different from MHC-II upregulation in pancreatic β-cells in human T1DM which was induced by IFN-γ stimulation (38, 39), which perhaps contribute to autoimmune response and β-cells dysfunction and in T1DM.

Taken together, we conclude that low MHC-II expression in inflammatory MC subsets features T2DM immune modulation and determines its lower Ag-presenting capacity. Therefore, we propose that reduced MHC-II levels in inflammatory MCs are an indicator or biomarker for severity and susceptibility to diabetes, inflammation, and suppression of Treg-dependent tolerance, especially in T2DM.

### Transcriptional signaling determines elevated inflammatory features in T1/2DM, and reduced Ag-presenting capacity in T2DM in Ly6C^high^ MCs

We identified 17 transcriptional axes via matching the diabetes-altered TFs and immunological genes in Ly6C^high^ MCs (**Figure 7B**) and considered that three transcriptional axes ↓PAX5-↓CIITA-↓Cd74/H2-Eb2, ↓MYC-↑Sema4a, and ↑CEBPA/E-↑Csf2ra/3r are most likely responsible for its reduced Ag-presenting capacity, and increased intercellular/intracellular inflammatory capacity.

PAX5 is a member of the paired box (Pax) family of TF and is critical for B-cell lineage commitment. We have identified similar PAX5 reduction in Ly6C^high^ MCs in HHcy and HL mice (17, 18) and proposed that Pax5 reduction (36.2-fold in control, 56.2-fold in *Cbs*^−/−^, and 10.9-fold in ApoE^−/−^ Ly6C^high^ MC) may modulate its corresponding genes of checkpoint (↑Sema4a/↓Tnfrsf9) and cytokine (↑Fas, ↓Il5ra/Il17rb) in Ly6C^high^ from both mice (17, 18). In this study, we found that the ↓PAX5-↓CIITA-↓Cd74/↓H2-Eb2 axis was suppressed in T2DM Ly6C^high^ MCs suggesting a reduced Ag-presenting capacity. This finding is well aligned with data presented in **Figure 3** showing that 10 MHC-II molecules downregulated in Ly6C^high^ MCs compared with those in Ly6C^low^ MCs. CD74 is an MHC-II invariant chain protein and is well known for its essential role in Ag presentation by mediating the assembly and subcellular trafficking of the MHC-II complex (40, 41). H2-Eb2 is involved in Ag processing and Ag peptide assembling and presentation, and positive regulation of T-cell activation (18). Taken together, we conclude that the Ag-presenting capacity is lower in Ly6C^high^ MCs than in Ly6C^low^ MCs in all mice and further suppressed in T2DM and that the ↓PAX5-↓CIITA-↓Cd74/↓H2-Eb2 axis may be a key mechanism for MHC-II suppression in diabetes, which is potentially responsible for T/B cell suppression and fibrotic/pro-inflammatory response identified in human and mouse diabetes (4, 5).

MYC is traditionally considered a transactivating TF associated with proliferative signaling and immune surveillance (42) and is also reported to suppress TGF-β (43), which usually maintains the quiescence and controls the activation of naive T cells and drives pro-inflammatory responses (44). We found that MYC was reduced 25-fold (log_2_FC 4.66) in T1DM and 10-fold (log_2_FC 3.32) in T2DM in Ly6C^high^ MCs compared with that in Ly6C^low^ MCs in the same mice, which matched with *Tgfb* induction in T1DM (2.5-fold, log_2_FC 1.32). Therefore, the identified ↓MYC-↑Sema4a axis, jointly with the ↓PAX5-↓CIITA-↓Cd74/H2-Eb2 axis, may contribute to intercellular inflammatory propagation capacity identified in human diabetes (4, 5) by Ly6C^high^ MC-derived MHC-II suppression and Sema4a induction.

Moreover, we found CEBPA/E induction in diabetic Ly6C^high^ MC. The corresponding activation of ↑CebpA-↑*Csf2ra* (T2DM) and ↑CebpA/CebpE-↑*Csf3r* (T1/2DM) axes underscore CSF2RA/CSF3R activation-related MC intracellular inflammatory capacity as we proposed above based on the fact that immune checkpoint and cytokine receptor expression determines intrinsic regulatory feedback in MC, allowing MCs to modulate their own activity and inflammatory features. This theory is consistent with the literature in that CSF3R (colony-stimulating factor 3 receptor) activated JAK-STAT and MAPK signaling and promoted myeloid cell proliferation and inflammation (45, 46) and that CSF2RA (colony stimulating factor 2 receptor subunit alpha) facilitated myeloid cell survival and pro-inflammatory cytokine production (47).

Our findings featured three critical transcriptional axes in diabetic Ly6C^high^ MCs each emphasizing a distinct immune function. The ↓PAX5-↓CIITA-↓CD74 is predominant in T2DM and impairs MHC-II assembly and Ag-presenting capacity. The ↓MYC-↑Sema4a axis is shared in T1/T2DM and is responsible for intercellular inflammatory propagation. The ↑CEBPA/E-↑CSF2R/CSF3R axis is predominant in T1DM and drives the MC intracellular inflammatory capacity.

### Clinical and functional implications

These transcriptomic signatures effectively recapitulate the distinct pathophysiological features of diabetes, translating into specific clinical consequences. In T2DM, the suppressed intercellular inflammatory propagation capacity—evidenced by the downregulation of the PAX5-CIITA regulatory axis and the subsequent attenuation of MHC-II and co-stimulatory ligands in Ly6Chigh MC—provides a robust molecular rationale for clinical immune paresis, encompassing impaired immune surveillance, heightened infection susceptibility, and delayed tissue repair. Conversely, the sustained type I IFN signaling and amplified intracellular inflammatory capacity uniquely observed in T1DM Ly6Chigh MCs drive a cell-autonomous, chronic inflammatory state that actively exacerbates systemic autoimmune complications and vascular injury. Ultimately, these findings suggest that therapeutically targeting these distinct, disease-specific MC-driven pathways could offer a precision medicine approach to mitigating diverse diabetic complications.

## Conclusions

We have three major discoveries: 1) Ly6C^high^ MCs exhibit lower Ag-presenting power, which was further suppressed in T2DM, mostly via ↓PAX5-↓CIITA-↓CD74 regulation. 2) Ly6C^high^ MCs display high intercellular inflammatory propagation but low intracellular inflammatory capacity, both further enhanced in T1/T2DM, mostly via ↓MYC-↑Sema4a and ↑CEBPA/E-↑Csf2ra/3r regulation. 3) Ly6C^low^ MCs acquired additional intercellular and intracellular inflammatory capacity in T1/T2DM.

## Limitations and progress

Transcriptome information of RNA-seq usually is generated from a great number of cells and provides in-depth genetic information and more sensitivity for the identification of novel molecular mechanisms. Further studies are needed to confirm the functional implications of the identified molecules and signaling in MC pathogenesis in various disease models. Nevertheless, the strong consensus between our bulk RNA-seq and independent scRNA-seq datasets provides robust, orthogonal evidence for the identified “intracellular inflammatory capacity” transcriptomic shifts.

## Data Availability

The data present in the study are deposited in the Gene Expression Omnibus (GEO) repository under the accession number GEO: GSE305525. The names of the repository/repositories and accession number(s) can be found in the article/**Supplementary Material**.

## Glossary

Ag: antigen
CD40: cluster of differentiation 40
CKD: chronic kidney disease
HHcy: hyperhomocysteinemia
MADS: metabolic-associated danger signal
Ag: antigen
MC: monocyte
Mϕ: macrophage
Ly6C: lymphocyte antigen 6 complex locus C
PC: principal components
PCA: principal components analysis
IPA: ingenuity pathway analysis
DEG: differentially expressed gene
RNA-seq: RNA-sequencing
scRNA-Seq: single-cell RNA sequencing
TF: transcriptional factor
STZ: streptozotocin
T1DM: type 1 diabetes mellitus
T2DM: Type 2 diabetes mellitus
CT: control
FC: fold change
FACS: fluorescent-activated cell sorting
AHR: aryl hydrocarbon receptor
PXR: pregnane X receptor
iNOS: inducible nitric oxide synthase
NFAT: nuclear factor of activated T-cells
PKCθ: protein kinase C theta
DC: dendritic cell
NK: natural killer
CAR: constitutive androstane receptor
PRRs: pattern recognition receptors
SLE: systemic lupus erythematosus
HMGB1: high mobility group box-1
THOP1: thimet oligopeptidase 1
Th1: T helper 1
IL-7: Interleukin 7
CHK: Csk-homologous kinase
NO: nitric oxide
ROS: reactive oxygen species
GF: growth factors.
