# Supplementary material for: Empowered pro-inflammatory features in Ly6C monocytes and altered antigen-presenting capacity in Ly6Chigh monocytes in diabetic mice
Source: Front Immunol. 2026 May 8;17:1794704. doi: 10.3389/fimmu.2026.1794704 (PMC13193908; doi:10.3389/fimmu.2026.1794704)
Supplement: Supplementary file 1 [file Presentation1.pptx]

## Slide 1
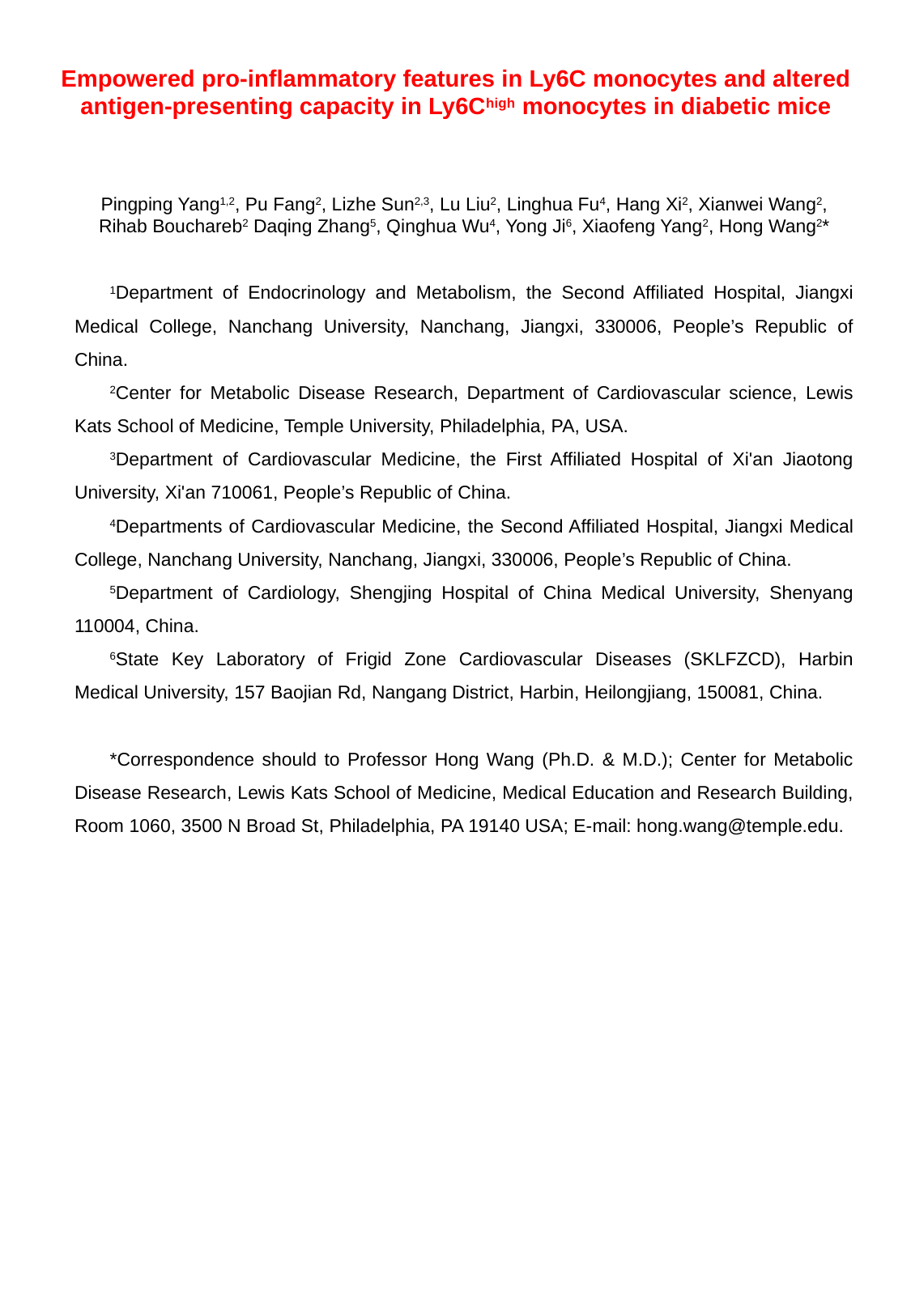

Empowered pro-inflammatory features in Ly6C monocytes and altered antigen-presenting capacity in Ly6Chigh monocytes in diabetic mice
Pingping Yang1,2, Pu Fang2, Lizhe Sun2,3, Lu Liu2, Linghua Fu4, Hang Xi2, Xianwei Wang2, Rihab Bouchareb2 Daqing Zhang5, Qinghua Wu4, Yong Ji6, Xiaofeng Yang2, Hong Wang2*
1Department of Endocrinology and Metabolism, the Second Affiliated Hospital, Jiangxi Medical College, Nanchang University, Nanchang, Jiangxi, 330006, People’s Republic of China.
2Center for Metabolic Disease Research, Department of Cardiovascular science, Lewis Kats School of Medicine, Temple University, Philadelphia, PA, USA.
3Department of Cardiovascular Medicine, the First Affiliated Hospital of Xi'an Jiaotong University, Xi'an 710061, People’s Republic of China.
4Departments of Cardiovascular Medicine, the Second Affiliated Hospital, Jiangxi Medical College, Nanchang University, Nanchang, Jiangxi, 330006, People’s Republic of China.
5Department of Cardiology, Shengjing Hospital of China Medical University, Shenyang 110004, China.
6State Key Laboratory of Frigid Zone Cardiovascular Diseases (SKLFZCD), Harbin Medical University, 157 Baojian Rd, Nangang District, Harbin, Heilongjiang, 150081, China.
*Correspondence should to Professor Hong Wang (Ph.D. & M.D.); Center for Metabolic Disease Research, Lewis Kats School of Medicine, Medical Education and Research Building, Room 1060, 3500 N Broad St, Philadelphia, PA 19140 USA; E-mail: hong.wang@temple.edu.

## Slide 2
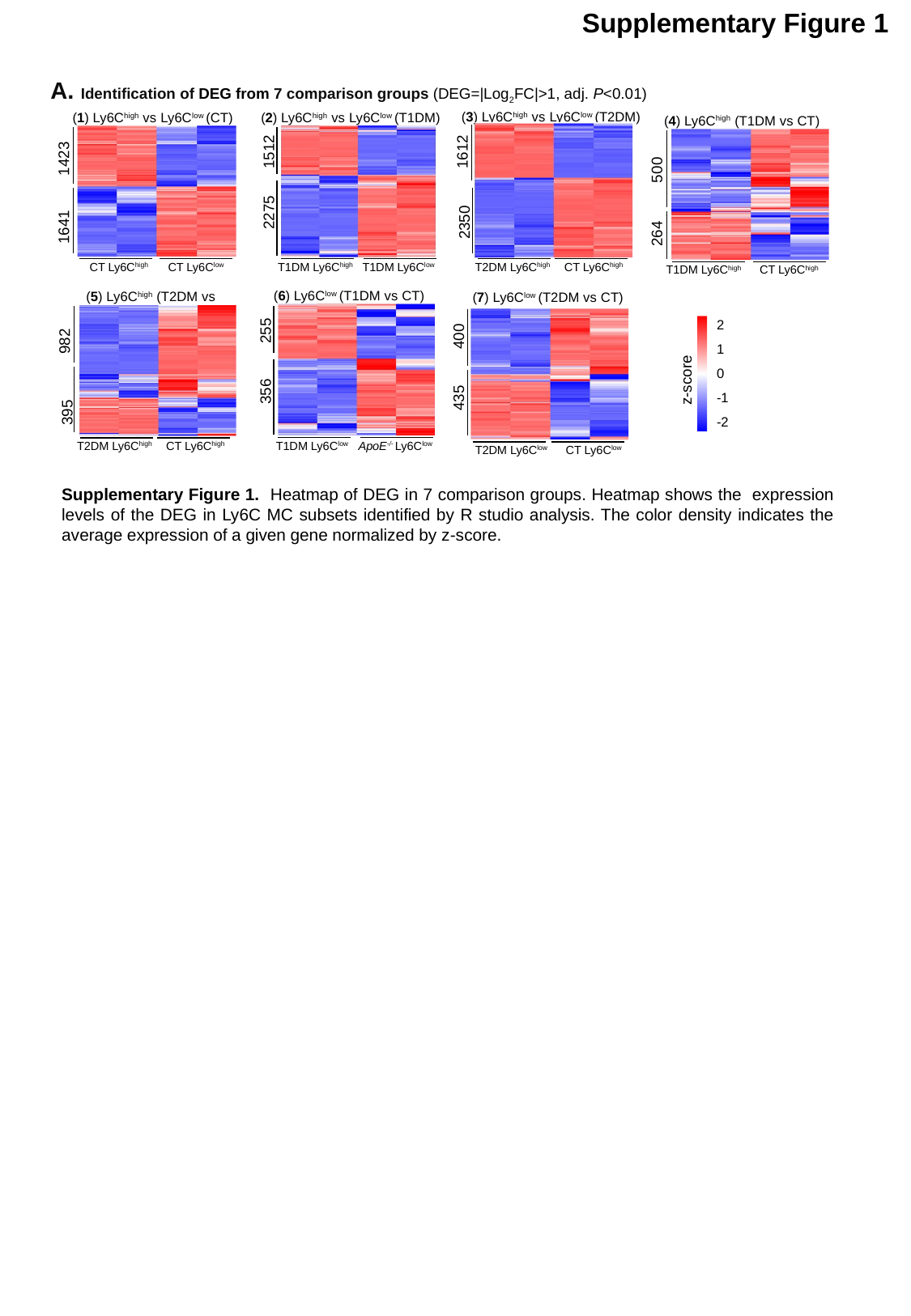

Supplementary Figure 1
A. Identification of DEG from 7 comparison groups (DEG=|Log2FC|>1, adj. P<0.01)
(3) Ly6Chigh vs Ly6Clow (T2DM)
(1) Ly6Chigh vs Ly6Clow (CT)
1423
1641
CT Ly6Chigh
CT Ly6Clow
(2) Ly6Chigh vs Ly6Clow (T1DM)
1512
2275
T1DM Ly6Chigh
T1DM Ly6Clow
(4) Ly6Chigh (T1DM vs CT)
1612
2350
T2DM Ly6Chigh
CT Ly6Chigh
500
264
T1DM Ly6Chigh
CT Ly6Chigh
(6) Ly6Clow (T1DM vs CT)
255
356
T1DM Ly6Clow
ApoE-/- Ly6Clow
(5) Ly6Chigh (T2DM vs CT)
982
395
T2DM Ly6Chigh
CT Ly6Chigh
(7) Ly6Clow (T2DM vs CT)
2
1
0
-1
-2
z-score
400
435
T2DM Ly6Clow
CT Ly6Clow
Supplementary Figure 1. Heatmap of DEG in 7 comparison groups. Heatmap shows the expression levels of the DEG in Ly6C MC subsets identified by R studio analysis. The color density indicates the average expression of a given gene normalized by z-score.

## Slide 3
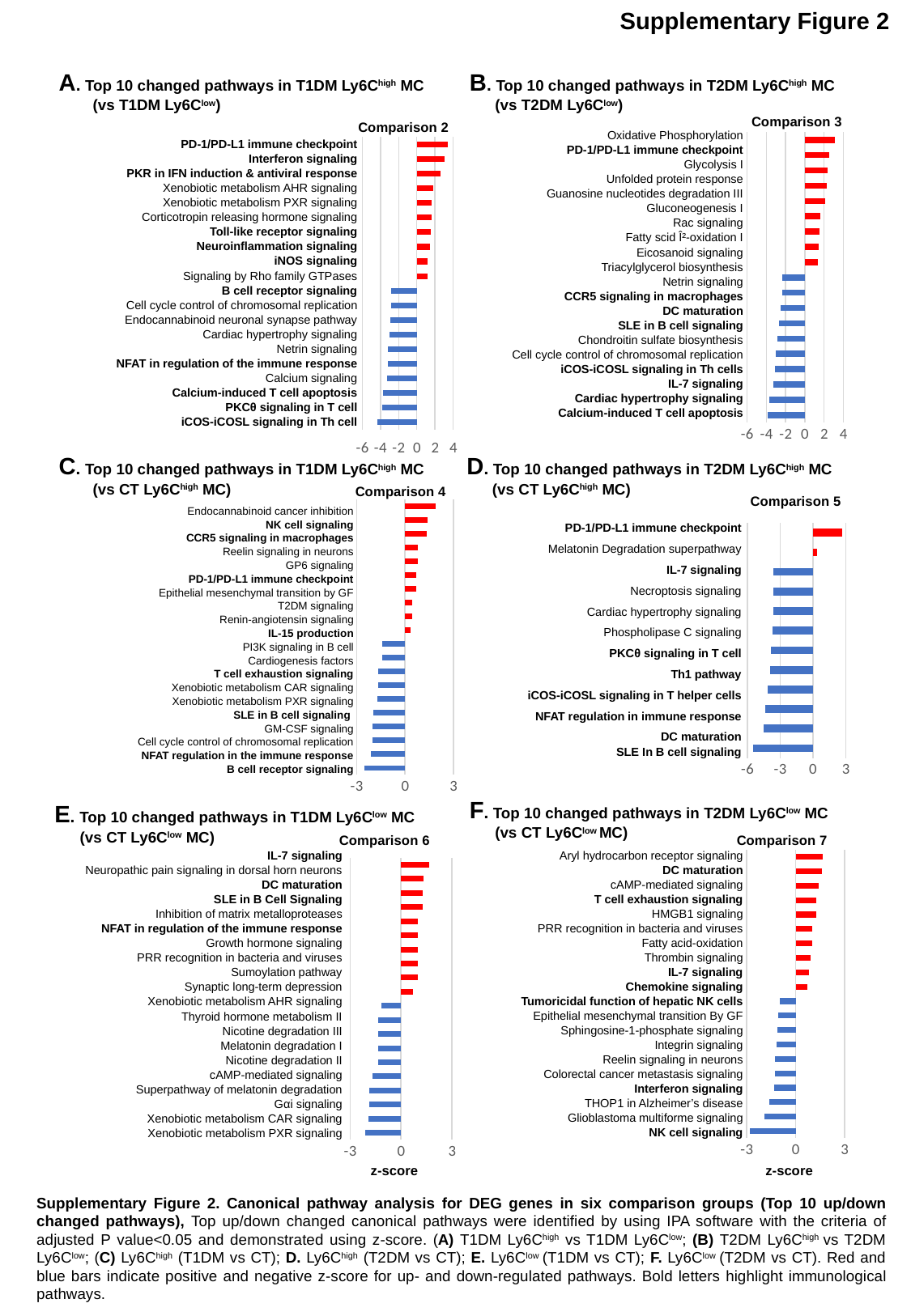

Supplementary Figure 2
A. Top 10 changed pathways in T1DM Ly6Chigh MC
 (vs T1DM Ly6Clow)
B. Top 10 changed pathways in T2DM Ly6Chigh MC
 (vs T2DM Ly6Clow)
Comparison 3
Comparison 2
### Chart
| Category | |
|---|---|
| iCOS-iCOSL Signaling in T Helper Cells | -4.382 |
| PKCθ Signaling in T Lymphocytes | -3.781 |
| Calcium-induced T Lymphocyte Apoptosis | -3.71 |
| Calcium Signaling | -3.272 |
| Role of NFAT in Regulation of the Immune Response | -3.175 |
| Netrin Signaling | -3.153 |
| Cardiac Hypertrophy Signaling (Enhanced) | -2.969 |
| Endocannabinoid Neuronal Synapse Pathway | -2.921 |
| Cell Cycle Control of Chromosomal Replication | -2.84 |
| B Cell Receptor Signaling | -2.795 |
| Signaling by Rho Family GTPases | 1.151 |
| iNOS Signaling | 1.155 |
| Neuroinflammation Signaling Pathway | 1.477 |
| Toll-like Receptor Signaling | 1.508 |
| Corticotropin Releasing Hormone Signaling | 1.633 |
| Xenobiotic Metabolism PXR Signaling Pathway | 1.64 |
| Xenobiotic Metabolism AHR Signaling Pathway | 1.789 |
| Role of PKR in Interferon Induction and Antiviral Response | 2.6 |
| Interferon Signaling | 3.051 |
| PD-1, PD-L1 cancer immunotherapy pathway | 3.402 |
### Chart
| Category | |
|---|---|| Oxidative Phosphorylation |
| --- |
| PD-1/PD-L1 immune checkpoint |
| Glycolysis I |
| Unfolded protein response |
| Guanosine nucleotides degradation III |
| Gluconeogenesis I |
| Rac signaling |
| Fatty scid Î²-oxidation I |
| Eicosanoid signaling |
| Triacylglycerol biosynthesis |
| Netrin signaling |
| CCR5 signaling in macrophages |
| DC maturation |
| SLE in B cell signaling |
| Chondroitin sulfate biosynthesis |
| Cell cycle control of chromosomal replication |
| iCOS-iCOSL signaling in Th cells |
| IL-7 signaling |
| Cardiac hypertrophy signaling |
| Calcium-induced T cell apoptosis |
| PD-1/PD-L1 immune checkpoint |
| --- |
| Interferon signaling |
| PKR in IFN induction & antiviral response |
| Xenobiotic metabolism AHR signaling |
| Xenobiotic metabolism PXR signaling |
| Corticotropin releasing hormone signaling |
| Toll-like receptor signaling |
| Neuroinflammation signaling |
| iNOS signaling |
| Signaling by Rho family GTPases |
| B cell receptor signaling |
| Cell cycle control of chromosomal replication |
| Endocannabinoid neuronal synapse pathway |
| Cardiac hypertrophy signaling |
| Netrin signaling |
| NFAT in regulation of the immune response |
| Calcium signaling |
| Calcium-induced T cell apoptosis |
| PKCθ signaling in T cell |
| iCOS-iCOSL signaling in Th cell |
C. Top 10 changed pathways in T1DM Ly6Chigh MC
 (vs CT Ly6Chigh MC)
D. Top 10 changed pathways in T2DM Ly6Chigh MC
 (vs CT Ly6Chigh MC)
Comparison 4
### Chart
| Category | |
|---|---|Comparison 5
### Chart
| Category | |
|---|---|| Endocannabinoid cancer inhibition |
| --- |
| NK cell signaling |
| CCR5 signaling in macrophages |
| Reelin signaling in neurons |
| GP6 signaling |
| PD-1/PD-L1 immune checkpoint |
| Epithelial mesenchymal transition by GF |
| T2DM signaling |
| Renin-angiotensin signaling |
| IL-15 production |
| PI3K signaling in B cell |
| Cardiogenesis factors |
| T cell exhaustion signaling |
| Xenobiotic metabolism CAR signaling |
| Xenobiotic metabolism PXR signaling |
| SLE in B cell signaling |
| GM-CSF signaling |
| Cell cycle control of chromosomal replication |
| NFAT regulation in the immune response |
| B cell receptor signaling |
| PD-1/PD-L1 immune checkpoint |
| --- |
| Melatonin Degradation superpathway |
| IL-7 signaling |
| Necroptosis signaling |
| Cardiac hypertrophy signaling |
| Phospholipase C signaling |
| PKCθ signaling in T cell |
| Th1 pathway |
| iCOS-iCOSL signaling in T helper cells |
| NFAT regulation in immune response |
| DC maturation |
| SLE In B cell signaling |
F. Top 10 changed pathways in T2DM Ly6Clow MC
 (vs CT Ly6Clow MC)
E. Top 10 changed pathways in T1DM Ly6Clow MC
 (vs CT Ly6Clow MC)
Comparison 6
Comparison 7
### Chart
| Category | |
|---|---|
### Chart
| Category | |
|---|---|| IL-7 signaling |
| --- |
| Neuropathic pain signaling in dorsal horn neurons |
| DC maturation |
| SLE in B Cell Signaling |
| Inhibition of matrix metalloproteases |
| NFAT in regulation of the immune response |
| Growth hormone signaling |
| PRR recognition in bacteria and viruses |
| Sumoylation pathway |
| Synaptic long-term depression |
| Xenobiotic metabolism AHR signaling |
| Thyroid hormone metabolism II |
| Nicotine degradation III |
| Melatonin degradation I |
| Nicotine degradation II |
| cAMP-mediated signaling |
| Superpathway of melatonin degradation |
| Gαi signaling |
| Xenobiotic metabolism CAR signaling |
| Xenobiotic metabolism PXR signaling |
| Aryl hydrocarbon receptor signaling |
| --- |
| DC maturation |
| cAMP-mediated signaling |
| T cell exhaustion signaling |
| HMGB1 signaling |
| PRR recognition in bacteria and viruses |
| Fatty acid-oxidation |
| Thrombin signaling |
| IL-7 signaling |
| Chemokine signaling |
| Tumoricidal function of hepatic NK cells |
| Epithelial mesenchymal transition By GF |
| Sphingosine-1-phosphate signaling |
| Integrin signaling |
| Reelin signaling in neurons |
| Colorectal cancer metastasis signaling |
| Interferon signaling |
| THOP1 in Alzheimer’s disease |
| Glioblastoma multiforme signaling |
| NK cell signaling |
z-score
z-score
Supplementary Figure 2. Canonical pathway analysis for DEG genes in six comparison groups (Top 10 up/down changed pathways), Top up/down changed canonical pathways were identified by using IPA software with the criteria of adjusted P value<0.05 and demonstrated using z-score. (A) T1DM Ly6Chigh vs T1DM Ly6Clow; (B) T2DM Ly6Chigh vs T2DM Ly6Clow; (C) Ly6Chigh (T1DM vs CT); D. Ly6Chigh (T2DM vs CT); E. Ly6Clow (T1DM vs CT); F. Ly6Clow (T2DM vs CT). Red and blue bars indicate positive and negative z-score for up- and down-regulated pathways. Bold letters highlight immunological pathways.

## Slide 4
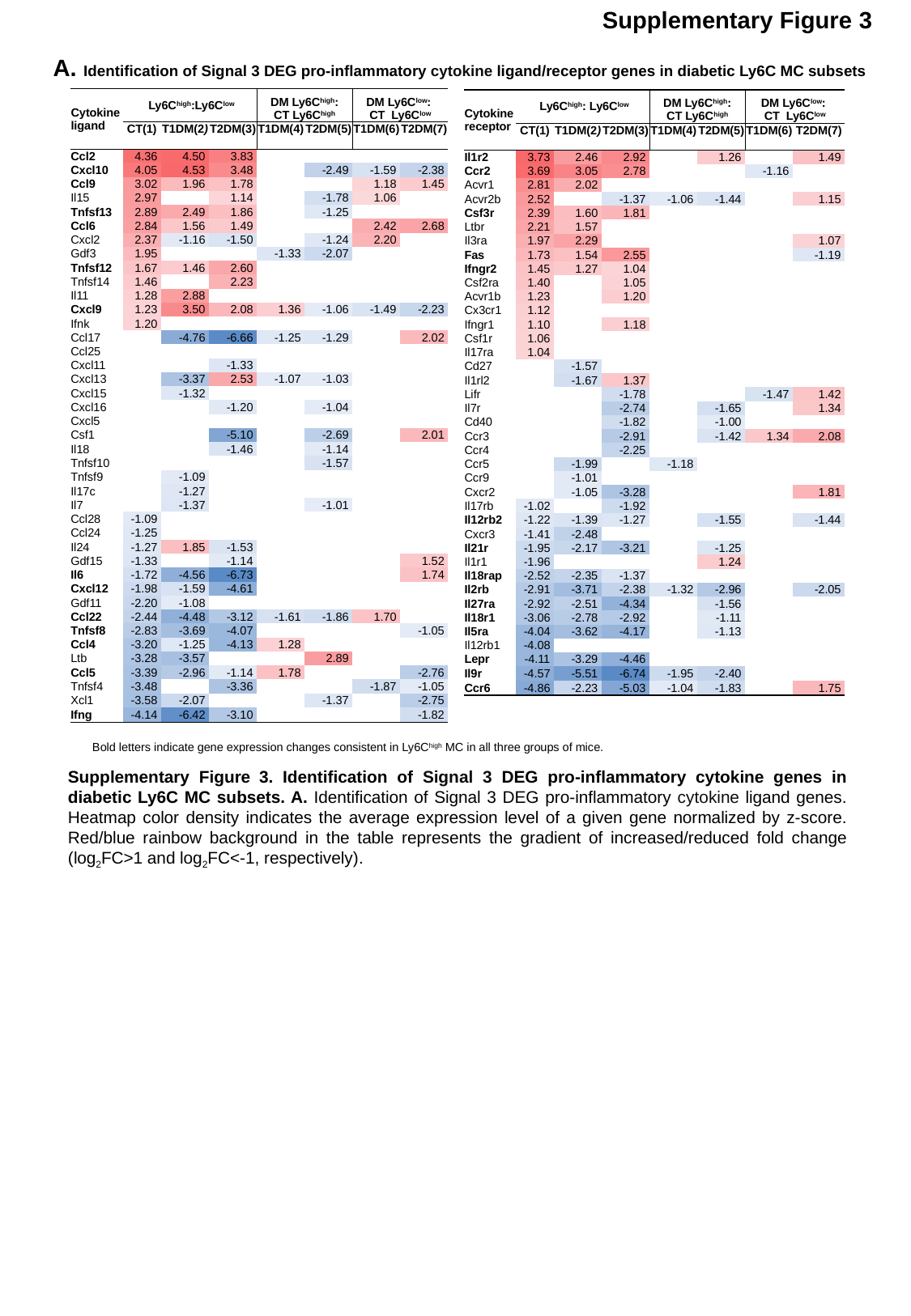

Supplementary Figure 3
A. Identification of Signal 3 DEG pro-inflammatory cytokine ligand/receptor genes in diabetic Ly6C MC subsets
| Cytokine ligand | Ly6Chigh:Ly6Clow | | | DM Ly6Chigh: CT Ly6Chigh | | DM Ly6Clow: CT Ly6Clow | |
| --- | --- | --- | --- | --- | --- | --- | --- |
| | CT(1) | T1DM(2) | T2DM(3) | T1DM(4) | T2DM(5) | T1DM(6) | T2DM(7) |
| Ccl2 | 4.36 | 4.50 | 3.83 | | | | |
| Cxcl10 | 4.05 | 4.53 | 3.48 | | -2.49 | -1.59 | -2.38 |
| Ccl9 | 3.02 | 1.96 | 1.78 | | | 1.18 | 1.45 |
| Il15 | 2.97 | | 1.14 | | -1.78 | 1.06 | |
| Tnfsf13 | 2.89 | 2.49 | 1.86 | | -1.25 | | |
| Ccl6 | 2.84 | 1.56 | 1.49 | | | 2.42 | 2.68 |
| Cxcl2 | 2.37 | -1.16 | -1.50 | | -1.24 | 2.20 | |
| Gdf3 | 1.95 | | | -1.33 | -2.07 | | |
| Tnfsf12 | 1.67 | 1.46 | 2.60 | | | | |
| Tnfsf14 | 1.46 | | 2.23 | | | | |
| Il11 | 1.28 | 2.88 | | | | | |
| Cxcl9 | 1.23 | 3.50 | 2.08 | 1.36 | -1.06 | -1.49 | -2.23 |
| Ifnk | 1.20 | | | | | | |
| Ccl17 | | -4.76 | -6.66 | -1.25 | -1.29 | | 2.02 |
| Ccl25 | | | | | | | |
| Cxcl11 | | | -1.33 | | | | |
| Cxcl13 | | -3.37 | 2.53 | -1.07 | -1.03 | | |
| Cxcl15 | | -1.32 | | | | | |
| Cxcl16 | | | -1.20 | | -1.04 | | |
| Cxcl5 | | | | | | | |
| Csf1 | | | -5.10 | | -2.69 | | 2.01 |
| Il18 | | | -1.46 | | -1.14 | | |
| Tnfsf10 | | | | | -1.57 | | |
| Tnfsf9 | | -1.09 | | | | | |
| Il17c | | -1.27 | | | | | |
| Il7 | | -1.37 | | | -1.01 | | |
| Ccl28 | -1.09 | | | | | | |
| Ccl24 | -1.25 | | | | | | |
| Il24 | -1.27 | 1.85 | -1.53 | | | | |
| Gdf15 | -1.33 | | -1.14 | | | | 1.52 |
| Il6 | -1.72 | -4.56 | -6.73 | | | | 1.74 |
| Cxcl12 | -1.98 | -1.59 | -4.61 | | | | |
| Gdf11 | -2.20 | -1.08 | | | | | |
| Ccl22 | -2.44 | -4.48 | -3.12 | -1.61 | -1.86 | 1.70 | |
| Tnfsf8 | -2.83 | -3.69 | -4.07 | | | | -1.05 |
| Ccl4 | -3.20 | -1.25 | -4.13 | 1.28 | | | |
| Ltb | -3.28 | -3.57 | | | 2.89 | | |
| Ccl5 | -3.39 | -2.96 | -1.14 | 1.78 | | | -2.76 |
| Tnfsf4 | -3.48 | | -3.36 | | | -1.87 | -1.05 |
| Xcl1 | -3.58 | -2.07 | | | -1.37 | | -2.75 |
| Ifng | -4.14 | -6.42 | -3.10 | | | | -1.82 |
| Cytokine receptor | Ly6Chigh: Ly6Clow | | | DM Ly6Chigh: CT Ly6Chigh | | DM Ly6Clow: CT Ly6Clow | |
| --- | --- | --- | --- | --- | --- | --- | --- |
| | CT(1) | T1DM(2) | T2DM(3) | T1DM(4) | T2DM(5) | T1DM(6) | T2DM(7) |
| Il1r2 | 3.73 | 2.46 | 2.92 | | 1.26 | | 1.49 |
| Ccr2 | 3.69 | 3.05 | 2.78 | | | -1.16 | |
| Acvr1 | 2.81 | 2.02 | | | | | |
| Acvr2b | 2.52 | | -1.37 | -1.06 | -1.44 | | 1.15 |
| Csf3r | 2.39 | 1.60 | 1.81 | | | | |
| Ltbr | 2.21 | 1.57 | | | | | |
| Il3ra | 1.97 | 2.29 | | | | | 1.07 |
| Fas | 1.73 | 1.54 | 2.55 | | | | -1.19 |
| Ifngr2 | 1.45 | 1.27 | 1.04 | | | | |
| Csf2ra | 1.40 | | 1.05 | | | | |
| Acvr1b | 1.23 | | 1.20 | | | | |
| Cx3cr1 | 1.12 | | | | | | |
| Ifngr1 | 1.10 | | 1.18 | | | | |
| Csf1r | 1.06 | | | | | | |
| Il17ra | 1.04 | | | | | | |
| Cd27 | | -1.57 | | | | | |
| Il1rl2 | | -1.67 | 1.37 | | | | |
| Lifr | | | -1.78 | | | -1.47 | 1.42 |
| Il7r | | | -2.74 | | -1.65 | | 1.34 |
| Cd40 | | | -1.82 | | -1.00 | | |
| Ccr3 | | | -2.91 | | -1.42 | 1.34 | 2.08 |
| Ccr4 | | | -2.25 | | | | |
| Ccr5 | | -1.99 | | -1.18 | | | |
| Ccr9 | | -1.01 | | | | | |
| Cxcr2 | | -1.05 | -3.28 | | | | 1.81 |
| Il17rb | -1.02 | | -1.92 | | | | |
| Il12rb2 | -1.22 | -1.39 | -1.27 | | -1.55 | | -1.44 |
| Cxcr3 | -1.41 | -2.48 | | | | | |
| Il21r | -1.95 | -2.17 | -3.21 | | -1.25 | | |
| Il1r1 | -1.96 | | | | 1.24 | | |
| Il18rap | -2.52 | -2.35 | -1.37 | | | | |
| Il2rb | -2.91 | -3.71 | -2.38 | -1.32 | -2.96 | | -2.05 |
| Il27ra | -2.92 | -2.51 | -4.34 | | -1.56 | | |
| Il18r1 | -3.06 | -2.78 | -2.92 | | -1.11 | | |
| Il5ra | -4.04 | -3.62 | -4.17 | | -1.13 | | |
| Il12rb1 | -4.08 | | | | | | |
| Lepr | -4.11 | -3.29 | -4.46 | | | | |
| Il9r | -4.57 | -5.51 | -6.74 | -1.95 | -2.40 | | |
| Ccr6 | -4.86 | -2.23 | -5.03 | -1.04 | -1.83 | | 1.75 |
Bold letters indicate gene expression changes consistent in Ly6Chigh MC in all three groups of mice.
Supplementary Figure 3. Identification of Signal 3 DEG pro-inflammatory cytokine genes in diabetic Ly6C MC subsets. A. Identification of Signal 3 DEG pro-inflammatory cytokine ligand genes. Heatmap color density indicates the average expression level of a given gene normalized by z-score. Red/blue rainbow background in the table represents the gradient of increased/reduced fold change (log2FC>1 and log2FC<-1, respectively).

## Slide 5
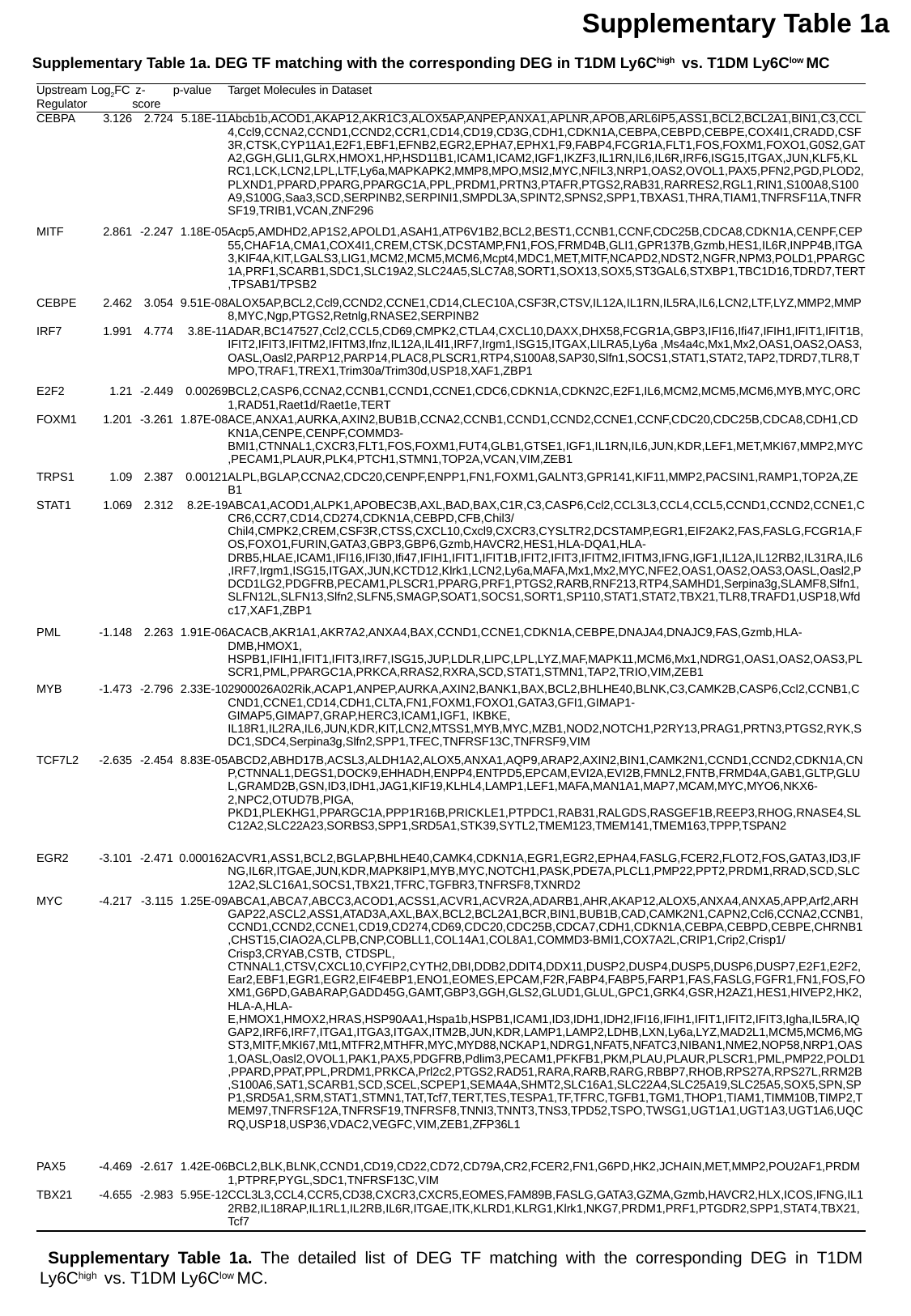

Supplementary Table 1a
Supplementary Table 1a. DEG TF matching with the corresponding DEG in T1DM Ly6Chigh vs. T1DM Ly6Clow MC
| Upstream Regulator | Log2FC | z-score | p-value | Target Molecules in Dataset |
| --- | --- | --- | --- | --- |
| CEBPA | 3.126 | 2.724 | 5.18E-11 | Abcb1b,ACOD1,AKAP12,AKR1C3,ALOX5AP,ANPEP,ANXA1,APLNR,APOB,ARL6IP5,ASS1,BCL2,BCL2A1,BIN1,C3,CCL4,Ccl9,CCNA2,CCND1,CCND2,CCR1,CD14,CD19,CD3G,CDH1,CDKN1A,CEBPA,CEBPD,CEBPE,COX4I1,CRADD,CSF3R,CTSK,CYP11A1,E2F1,EBF1,EFNB2,EGR2,EPHA7,EPHX1,F9,FABP4,FCGR1A,FLT1,FOS,FOXM1,FOXO1,G0S2,GATA2,GGH,GLI1,GLRX,HMOX1,HP,HSD11B1,ICAM1,ICAM2,IGF1,IKZF3,IL1RN,IL6,IL6R,IRF6,ISG15,ITGAX,JUN,KLF5,KLRC1,LCK,LCN2,LPL,LTF,Ly6a,MAPKAPK2,MMP8,MPO,MSI2,MYC,NFIL3,NRP1,OAS2,OVOL1,PAX5,PFN2,PGD,PLOD2,PLXND1,PPARD,PPARG,PPARGC1A,PPL,PRDM1,PRTN3,PTAFR,PTGS2,RAB31,RARRES2,RGL1,RIN1,S100A8,S100A9,S100G,Saa3,SCD,SERPINB2,SERPINI1,SMPDL3A,SPINT2,SPNS2,SPP1,TBXAS1,THRA,TIAM1,TNFRSF11A,TNFRSF19,TRIB1,VCAN,ZNF296 |
| MITF | 2.861 | -2.247 | 1.18E-05 | Acp5,AMDHD2,AP1S2,APOLD1,ASAH1,ATP6V1B2,BCL2,BEST1,CCNB1,CCNF,CDC25B,CDCA8,CDKN1A,CENPF,CEP55,CHAF1A,CMA1,COX4I1,CREM,CTSK,DCSTAMP,FN1,FOS,FRMD4B,GLI1,GPR137B,Gzmb,HES1,IL6R,INPP4B,ITGA3,KIF4A,KIT,LGALS3,LIG1,MCM2,MCM5,MCM6,Mcpt4,MDC1,MET,MITF,NCAPD2,NDST2,NGFR,NPM3,POLD1,PPARGC1A,PRF1,SCARB1,SDC1,SLC19A2,SLC24A5,SLC7A8,SORT1,SOX13,SOX5,ST3GAL6,STXBP1,TBC1D16,TDRD7,TERT,TPSAB1/TPSB2 |
| CEBPE | 2.462 | 3.054 | 9.51E-08 | ALOX5AP,BCL2,Ccl9,CCND2,CCNE1,CD14,CLEC10A,CSF3R,CTSV,IL12A,IL1RN,IL5RA,IL6,LCN2,LTF,LYZ,MMP2,MMP8,MYC,Ngp,PTGS2,Retnlg,RNASE2,SERPINB2 |
| IRF7 | 1.991 | 4.774 | 3.8E-11 | ADAR,BC147527,Ccl2,CCL5,CD69,CMPK2,CTLA4,CXCL10,DAXX,DHX58,FCGR1A,GBP3,IFI16,Ifi47,IFIH1,IFIT1,IFIT1B,IFIT2,IFIT3,IFITM2,IFITM3,Ifnz,IL12A,IL4I1,IRF7,Irgm1,ISG15,ITGAX,LILRA5,Ly6a ,Ms4a4c,Mx1,Mx2,OAS1,OAS2,OAS3,OASL,Oasl2,PARP12,PARP14,PLAC8,PLSCR1,RTP4,S100A8,SAP30,Slfn1,SOCS1,STAT1,STAT2,TAP2,TDRD7,TLR8,TMPO,TRAF1,TREX1,Trim30a/Trim30d,USP18,XAF1,ZBP1 |
| E2F2 | 1.21 | -2.449 | 0.00269 | BCL2,CASP6,CCNA2,CCNB1,CCND1,CCNE1,CDC6,CDKN1A,CDKN2C,E2F1,IL6,MCM2,MCM5,MCM6,MYB,MYC,ORC1,RAD51,Raet1d/Raet1e,TERT |
| FOXM1 | 1.201 | -3.261 | 1.87E-08 | ACE,ANXA1,AURKA,AXIN2,BUB1B,CCNA2,CCNB1,CCND1,CCND2,CCNE1,CCNF,CDC20,CDC25B,CDCA8,CDH1,CDKN1A,CENPE,CENPF,COMMD3-BMI1,CTNNAL1,CXCR3,FLT1,FOS,FOXM1,FUT4,GLB1,GTSE1,IGF1,IL1RN,IL6,JUN,KDR,LEF1,MET,MKI67,MMP2,MYC,PECAM1,PLAUR,PLK4,PTCH1,STMN1,TOP2A,VCAN,VIM,ZEB1 |
| TRPS1 | 1.09 | 2.387 | 0.00121 | ALPL,BGLAP,CCNA2,CDC20,CENPF,ENPP1,FN1,FOXM1,GALNT3,GPR141,KIF11,MMP2,PACSIN1,RAMP1,TOP2A,ZEB1 |
| STAT1 | 1.069 | 2.312 | 8.2E-19 | ABCA1,ACOD1,ALPK1,APOBEC3B,AXL,BAD,BAX,C1R,C3,CASP6,Ccl2,CCL3L3,CCL4,CCL5,CCND1,CCND2,CCNE1,CCR6,CCR7,CD14,CD274,CDKN1A,CEBPD,CFB,Chil3/Chil4,CMPK2,CREM,CSF3R,CTSS,CXCL10,Cxcl9,CXCR3,CYSLTR2,DCSTAMP,EGR1,EIF2AK2,FAS,FASLG,FCGR1A,FOS,FOXO1,FURIN,GATA3,GBP3,GBP6,Gzmb,HAVCR2,HES1,HLA-DQA1,HLA-DRB5,HLAE,ICAM1,IFI16,IFI30,Ifi47,IFIH1,IFIT1,IFIT1B,IFIT2,IFIT3,IFITM2,IFITM3,IFNG,IGF1,IL12A,IL12RB2,IL31RA,IL6,IRF7,Irgm1,ISG15,ITGAX,JUN,KCTD12,Klrk1,LCN2,Ly6a,MAFA,Mx1,Mx2,MYC,NFE2,OAS1,OAS2,OAS3,OASL,Oasl2,PDCD1LG2,PDGFRB,PECAM1,PLSCR1,PPARG,PRF1,PTGS2,RARB,RNF213,RTP4,SAMHD1,Serpina3g,SLAMF8,Slfn1,SLFN12L,SLFN13,Slfn2,SLFN5,SMAGP,SOAT1,SOCS1,SORT1,SP110,STAT1,STAT2,TBX21,TLR8,TRAFD1,USP18,Wfdc17,XAF1,ZBP1 |
| PML | -1.148 | 2.263 | 1.91E-06 | ACACB,AKR1A1,AKR7A2,ANXA4,BAX,CCND1,CCNE1,CDKN1A,CEBPE,DNAJA4,DNAJC9,FAS,Gzmb,HLA-DMB,HMOX1, HSPB1,IFIH1,IFIT1,IFIT3,IRF7,ISG15,JUP,LDLR,LIPC,LPL,LYZ,MAF,MAPK11,MCM6,Mx1,NDRG1,OAS1,OAS2,OAS3,PLSCR1,PML,PPARGC1A,PRKCA,RRAS2,RXRA,SCD,STAT1,STMN1,TAP2,TRIO,VIM,ZEB1 |
| MYB | -1.473 | -2.796 | 2.33E-10 | 2900026A02Rik,ACAP1,ANPEP,AURKA,AXIN2,BANK1,BAX,BCL2,BHLHE40,BLNK,C3,CAMK2B,CASP6,Ccl2,CCNB1,CCND1,CCNE1,CD14,CDH1,CLTA,FN1,FOXM1,FOXO1,GATA3,GFI1,GIMAP1-GIMAP5,GIMAP7,GRAP,HERC3,ICAM1,IGF1, IKBKE, IL18R1,IL2RA,IL6,JUN,KDR,KIT,LCN2,MTSS1,MYB,MYC,MZB1,NOD2,NOTCH1,P2RY13,PRAG1,PRTN3,PTGS2,RYK,SDC1,SDC4,Serpina3g,Slfn2,SPP1,TFEC,TNFRSF13C,TNFRSF9,VIM |
| TCF7L2 | -2.635 | -2.454 | 8.83E-05 | ABCD2,ABHD17B,ACSL3,ALDH1A2,ALOX5,ANXA1,AQP9,ARAP2,AXIN2,BIN1,CAMK2N1,CCND1,CCND2,CDKN1A,CNP,CTNNAL1,DEGS1,DOCK9,EHHADH,ENPP4,ENTPD5,EPCAM,EVI2A,EVI2B,FMNL2,FNTB,FRMD4A,GAB1,GLTP,GLUL,GRAMD2B,GSN,ID3,IDH1,JAG1,KIF19,KLHL4,LAMP1,LEF1,MAFA,MAN1A1,MAP7,MCAM,MYC,MYO6,NKX6-2,NPC2,OTUD7B,PIGA, PKD1,PLEKHG1,PPARGC1A,PPP1R16B,PRICKLE1,PTPDC1,RAB31,RALGDS,RASGEF1B,REEP3,RHOG,RNASE4,SLC12A2,SLC22A23,SORBS3,SPP1,SRD5A1,STK39,SYTL2,TMEM123,TMEM141,TMEM163,TPPP,TSPAN2 |
| EGR2 | -3.101 | -2.471 | 0.000162 | ACVR1,ASS1,BCL2,BGLAP,BHLHE40,CAMK4,CDKN1A,EGR1,EGR2,EPHA4,FASLG,FCER2,FLOT2,FOS,GATA3,ID3,IFNG,IL6R,ITGAE,JUN,KDR,MAPK8IP1,MYB,MYC,NOTCH1,PASK,PDE7A,PLCL1,PMP22,PPT2,PRDM1,RRAD,SCD,SLC12A2,SLC16A1,SOCS1,TBX21,TFRC,TGFBR3,TNFRSF8,TXNRD2 |
| MYC | -4.217 | -3.115 | 1.25E-09 | ABCA1,ABCA7,ABCC3,ACOD1,ACSS1,ACVR1,ACVR2A,ADARB1,AHR,AKAP12,ALOX5,ANXA4,ANXA5,APP,Arf2,ARHGAP22,ASCL2,ASS1,ATAD3A,AXL,BAX,BCL2,BCL2A1,BCR,BIN1,BUB1B,CAD,CAMK2N1,CAPN2,Ccl6,CCNA2,CCNB1,CCND1,CCND2,CCNE1,CD19,CD274,CD69,CDC20,CDC25B,CDCA7,CDH1,CDKN1A,CEBPA,CEBPD,CEBPE,CHRNB1,CHST15,CIAO2A,CLPB,CNP,COBLL1,COL14A1,COL8A1,COMMD3-BMI1,COX7A2L,CRIP1,Crip2,Crisp1/Crisp3,CRYAB,CSTB, CTDSPL, CTNNAL1,CTSV,CXCL10,CYFIP2,CYTH2,DBI,DDB2,DDIT4,DDX11,DUSP2,DUSP4,DUSP5,DUSP6,DUSP7,E2F1,E2F2,Ear2,EBF1,EGR1,EGR2,EIF4EBP1,ENO1,EOMES,EPCAM,F2R,FABP4,FABP5,FARP1,FAS,FASLG,FGFR1,FN1,FOS,FOXM1,G6PD,GABARAP,GADD45G,GAMT,GBP3,GGH,GLS2,GLUD1,GLUL,GPC1,GRK4,GSR,H2AZ1,HES1,HIVEP2,HK2,HLA-A,HLA-E,HMOX1,HMOX2,HRAS,HSP90AA1,Hspa1b,HSPB1,ICAM1,ID3,IDH1,IDH2,IFI16,IFIH1,IFIT1,IFIT2,IFIT3,Igha,IL5RA,IQGAP2,IRF6,IRF7,ITGA1,ITGA3,ITGAX,ITM2B,JUN,KDR,LAMP1,LAMP2,LDHB,LXN,Ly6a,LYZ,MAD2L1,MCM5,MCM6,MGST3,MITF,MKI67,Mt1,MTFR2,MTHFR,MYC,MYD88,NCKAP1,NDRG1,NFAT5,NFATC3,NIBAN1,NME2,NOP58,NRP1,OAS1,OASL,Oasl2,OVOL1,PAK1,PAX5,PDGFRB,Pdlim3,PECAM1,PFKFB1,PKM,PLAU,PLAUR,PLSCR1,PML,PMP22,POLD1,PPARD,PPAT,PPL,PRDM1,PRKCA,Prl2c2,PTGS2,RAD51,RARA,RARB,RARG,RBBP7,RHOB,RPS27A,RPS27L,RRM2B,S100A6,SAT1,SCARB1,SCD,SCEL,SCPEP1,SEMA4A,SHMT2,SLC16A1,SLC22A4,SLC25A19,SLC25A5,SOX5,SPN,SPP1,SRD5A1,SRM,STAT1,STMN1,TAT,Tcf7,TERT,TES,TESPA1,TF,TFRC,TGFB1,TGM1,THOP1,TIAM1,TIMM10B,TIMP2,TMEM97,TNFRSF12A,TNFRSF19,TNFRSF8,TNNI3,TNNT3,TNS3,TPD52,TSPO,TWSG1,UGT1A1,UGT1A3,UGT1A6,UQCRQ,USP18,USP36,VDAC2,VEGFC,VIM,ZEB1,ZFP36L1 |
| PAX5 | -4.469 | -2.617 | 1.42E-06 | BCL2,BLK,BLNK,CCND1,CD19,CD22,CD72,CD79A,CR2,FCER2,FN1,G6PD,HK2,JCHAIN,MET,MMP2,POU2AF1,PRDM1,PTPRF,PYGL,SDC1,TNFRSF13C,VIM |
| TBX21 | -4.655 | -2.983 | 5.95E-12 | CCL3L3,CCL4,CCR5,CD38,CXCR3,CXCR5,EOMES,FAM89B,FASLG,GATA3,GZMA,Gzmb,HAVCR2,HLX,ICOS,IFNG,IL12RB2,IL18RAP,IL1RL1,IL2RB,IL6R,ITGAE,ITK,KLRD1,KLRG1,Klrk1,NKG7,PRDM1,PRF1,PTGDR2,SPP1,STAT4,TBX21,Tcf7 |
 Supplementary Table 1a. The detailed list of DEG TF matching with the corresponding DEG in T1DM Ly6Chigh vs. T1DM Ly6Clow MC.

## Slide 6
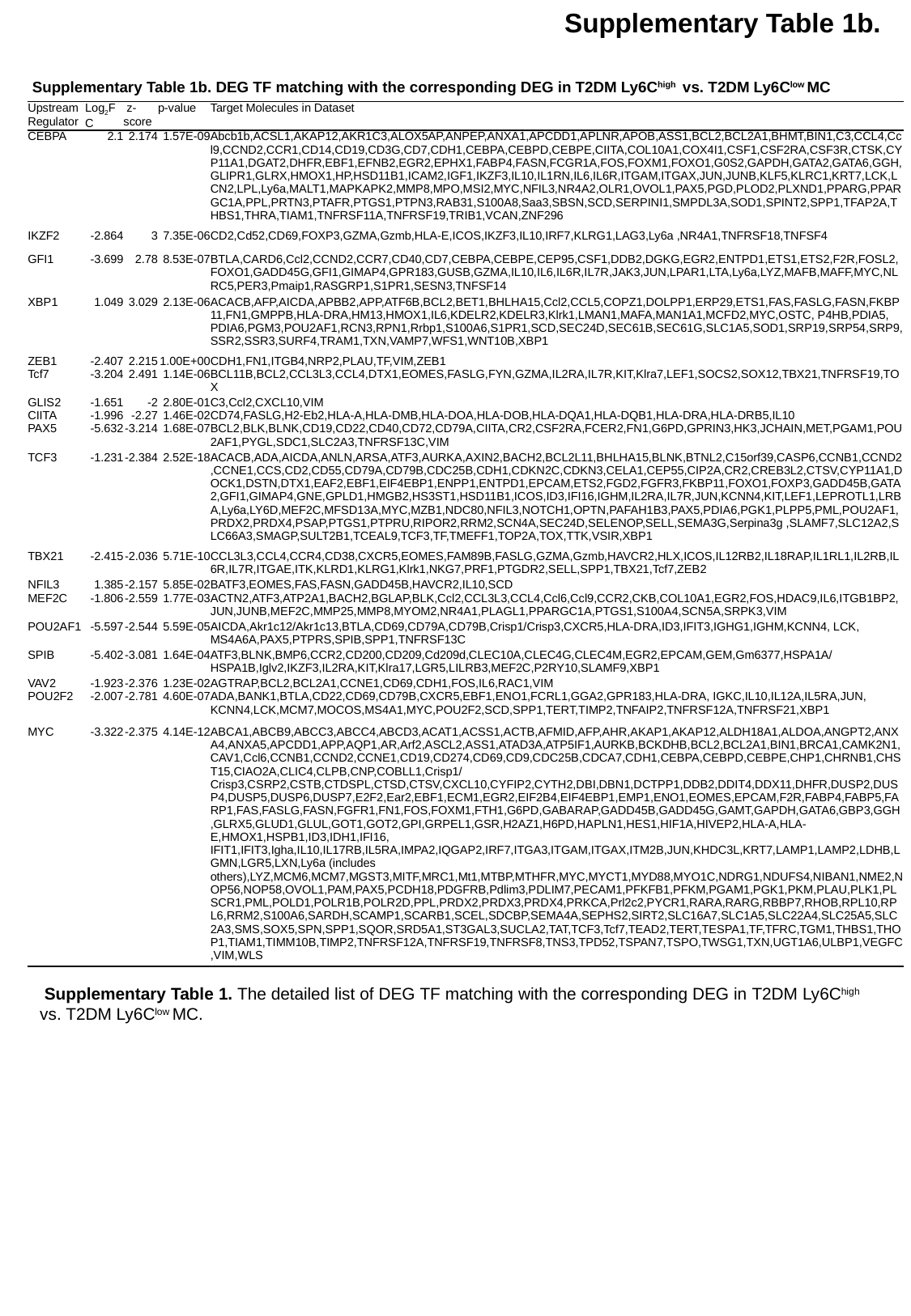

Supplementary Table 1b.
Supplementary Table 1b. DEG TF matching with the corresponding DEG in T2DM Ly6Chigh vs. T2DM Ly6Clow MC
| Upstream Regulator | Log2FC | z-score | p-value | Target Molecules in Dataset |
| --- | --- | --- | --- | --- |
| CEBPA | 2.1 | 2.174 | 1.57E-09 | Abcb1b,ACSL1,AKAP12,AKR1C3,ALOX5AP,ANPEP,ANXA1,APCDD1,APLNR,APOB,ASS1,BCL2,BCL2A1,BHMT,BIN1,C3,CCL4,Ccl9,CCND2,CCR1,CD14,CD19,CD3G,CD7,CDH1,CEBPA,CEBPD,CEBPE,CIITA,COL10A1,COX4I1,CSF1,CSF2RA,CSF3R,CTSK,CYP11A1,DGAT2,DHFR,EBF1,EFNB2,EGR2,EPHX1,FABP4,FASN,FCGR1A,FOS,FOXM1,FOXO1,G0S2,GAPDH,GATA2,GATA6,GGH,GLIPR1,GLRX,HMOX1,HP,HSD11B1,ICAM2,IGF1,IKZF3,IL10,IL1RN,IL6,IL6R,ITGAM,ITGAX,JUN,JUNB,KLF5,KLRC1,KRT7,LCK,LCN2,LPL,Ly6a,MALT1,MAPKAPK2,MMP8,MPO,MSI2,MYC,NFIL3,NR4A2,OLR1,OVOL1,PAX5,PGD,PLOD2,PLXND1,PPARG,PPARGC1A,PPL,PRTN3,PTAFR,PTGS1,PTPN3,RAB31,S100A8,Saa3,SBSN,SCD,SERPINI1,SMPDL3A,SOD1,SPINT2,SPP1,TFAP2A,THBS1,THRA,TIAM1,TNFRSF11A,TNFRSF19,TRIB1,VCAN,ZNF296 |
| IKZF2 | -2.864 | 3 | 7.35E-06 | CD2,Cd52,CD69,FOXP3,GZMA,Gzmb,HLA-E,ICOS,IKZF3,IL10,IRF7,KLRG1,LAG3,Ly6a ,NR4A1,TNFRSF18,TNFSF4 |
| GFI1 | -3.699 | 2.78 | 8.53E-07 | BTLA,CARD6,Ccl2,CCND2,CCR7,CD40,CD7,CEBPA,CEBPE,CEP95,CSF1,DDB2,DGKG,EGR2,ENTPD1,ETS1,ETS2,F2R,FOSL2,FOXO1,GADD45G,GFI1,GIMAP4,GPR183,GUSB,GZMA,IL10,IL6,IL6R,IL7R,JAK3,JUN,LPAR1,LTA,Ly6a,LYZ,MAFB,MAFF,MYC,NLRC5,PER3,Pmaip1,RASGRP1,S1PR1,SESN3,TNFSF14 |
| XBP1 | 1.049 | 3.029 | 2.13E-06 | ACACB,AFP,AICDA,APBB2,APP,ATF6B,BCL2,BET1,BHLHA15,Ccl2,CCL5,COPZ1,DOLPP1,ERP29,ETS1,FAS,FASLG,FASN,FKBP11,FN1,GMPPB,HLA-DRA,HM13,HMOX1,IL6,KDELR2,KDELR3,Klrk1,LMAN1,MAFA,MAN1A1,MCFD2,MYC,OSTC, P4HB,PDIA5, PDIA6,PGM3,POU2AF1,RCN3,RPN1,Rrbp1,S100A6,S1PR1,SCD,SEC24D,SEC61B,SEC61G,SLC1A5,SOD1,SRP19,SRP54,SRP9,SSR2,SSR3,SURF4,TRAM1,TXN,VAMP7,WFS1,WNT10B,XBP1 |
| ZEB1 | -2.407 | 2.215 | 1.00E+00 | CDH1,FN1,ITGB4,NRP2,PLAU,TF,VIM,ZEB1 |
| Tcf7 | -3.204 | 2.491 | 1.14E-06 | BCL11B,BCL2,CCL3L3,CCL4,DTX1,EOMES,FASLG,FYN,GZMA,IL2RA,IL7R,KIT,Klra7,LEF1,SOCS2,SOX12,TBX21,TNFRSF19,TOX |
| GLIS2 | -1.651 | -2 | 2.80E-01 | C3,Ccl2,CXCL10,VIM |
| CIITA | -1.996 | -2.27 | 1.46E-02 | CD74,FASLG,H2-Eb2,HLA-A,HLA-DMB,HLA-DOA,HLA-DOB,HLA-DQA1,HLA-DQB1,HLA-DRA,HLA-DRB5,IL10 |
| PAX5 | -5.632 | -3.214 | 1.68E-07 | BCL2,BLK,BLNK,CD19,CD22,CD40,CD72,CD79A,CIITA,CR2,CSF2RA,FCER2,FN1,G6PD,GPRIN3,HK3,JCHAIN,MET,PGAM1,POU2AF1,PYGL,SDC1,SLC2A3,TNFRSF13C,VIM |
| TCF3 | -1.231 | -2.384 | 2.52E-18 | ACACB,ADA,AICDA,ANLN,ARSA,ATF3,AURKA,AXIN2,BACH2,BCL2L11,BHLHA15,BLNK,BTNL2,C15orf39,CASP6,CCNB1,CCND2,CCNE1,CCS,CD2,CD55,CD79A,CD79B,CDC25B,CDH1,CDKN2C,CDKN3,CELA1,CEP55,CIP2A,CR2,CREB3L2,CTSV,CYP11A1,DOCK1,DSTN,DTX1,EAF2,EBF1,EIF4EBP1,ENPP1,ENTPD1,EPCAM,ETS2,FGD2,FGFR3,FKBP11,FOXO1,FOXP3,GADD45B,GATA2,GFI1,GIMAP4,GNE,GPLD1,HMGB2,HS3ST1,HSD11B1,ICOS,ID3,IFI16,IGHM,IL2RA,IL7R,JUN,KCNN4,KIT,LEF1,LEPROTL1,LRBA,Ly6a,LY6D,MEF2C,MFSD13A,MYC,MZB1,NDC80,NFIL3,NOTCH1,OPTN,PAFAH1B3,PAX5,PDIA6,PGK1,PLPP5,PML,POU2AF1,PRDX2,PRDX4,PSAP,PTGS1,PTPRU,RIPOR2,RRM2,SCN4A,SEC24D,SELENOP,SELL,SEMA3G,Serpina3g ,SLAMF7,SLC12A2,SLC66A3,SMAGP,SULT2B1,TCEAL9,TCF3,TF,TMEFF1,TOP2A,TOX,TTK,VSIR,XBP1 |
| TBX21 | -2.415 | -2.036 | 5.71E-10 | CCL3L3,CCL4,CCR4,CD38,CXCR5,EOMES,FAM89B,FASLG,GZMA,Gzmb,HAVCR2,HLX,ICOS,IL12RB2,IL18RAP,IL1RL1,IL2RB,IL6R,IL7R,ITGAE,ITK,KLRD1,KLRG1,Klrk1,NKG7,PRF1,PTGDR2,SELL,SPP1,TBX21,Tcf7,ZEB2 |
| NFIL3 | 1.385 | -2.157 | 5.85E-02 | BATF3,EOMES,FAS,FASN,GADD45B,HAVCR2,IL10,SCD |
| MEF2C | -1.806 | -2.559 | 1.77E-03 | ACTN2,ATF3,ATP2A1,BACH2,BGLAP,BLK,Ccl2,CCL3L3,CCL4,Ccl6,Ccl9,CCR2,CKB,COL10A1,EGR2,FOS,HDAC9,IL6,ITGB1BP2,JUN,JUNB,MEF2C,MMP25,MMP8,MYOM2,NR4A1,PLAGL1,PPARGC1A,PTGS1,S100A4,SCN5A,SRPK3,VIM |
| POU2AF1 | -5.597 | -2.544 | 5.59E-05 | AICDA,Akr1c12/Akr1c13,BTLA,CD69,CD79A,CD79B,Crisp1/Crisp3,CXCR5,HLA-DRA,ID3,IFIT3,IGHG1,IGHM,KCNN4, LCK, MS4A6A,PAX5,PTPRS,SPIB,SPP1,TNFRSF13C |
| SPIB | -5.402 | -3.081 | 1.64E-04 | ATF3,BLNK,BMP6,CCR2,CD200,CD209,Cd209d,CLEC10A,CLEC4G,CLEC4M,EGR2,EPCAM,GEM,Gm6377,HSPA1A/HSPA1B,Iglv2,IKZF3,IL2RA,KIT,Klra17,LGR5,LILRB3,MEF2C,P2RY10,SLAMF9,XBP1 |
| VAV2 | -1.923 | -2.376 | 1.23E-02 | AGTRAP,BCL2,BCL2A1,CCNE1,CD69,CDH1,FOS,IL6,RAC1,VIM |
| POU2F2 | -2.007 | -2.781 | 4.60E-07 | ADA,BANK1,BTLA,CD22,CD69,CD79B,CXCR5,EBF1,ENO1,FCRL1,GGA2,GPR183,HLA-DRA, IGKC,IL10,IL12A,IL5RA,JUN, KCNN4,LCK,MCM7,MOCOS,MS4A1,MYC,POU2F2,SCD,SPP1,TERT,TIMP2,TNFAIP2,TNFRSF12A,TNFRSF21,XBP1 |
| MYC | -3.322 | -2.375 | 4.14E-12 | ABCA1,ABCB9,ABCC3,ABCC4,ABCD3,ACAT1,ACSS1,ACTB,AFMID,AFP,AHR,AKAP1,AKAP12,ALDH18A1,ALDOA,ANGPT2,ANXA4,ANXA5,APCDD1,APP,AQP1,AR,Arf2,ASCL2,ASS1,ATAD3A,ATP5IF1,AURKB,BCKDHB,BCL2,BCL2A1,BIN1,BRCA1,CAMK2N1,CAV1,Ccl6,CCNB1,CCND2,CCNE1,CD19,CD274,CD69,CD9,CDC25B,CDCA7,CDH1,CEBPA,CEBPD,CEBPE,CHP1,CHRNB1,CHST15,CIAO2A,CLIC4,CLPB,CNP,COBLL1,Crisp1/Crisp3,CSRP2,CSTB,CTDSPL,CTSD,CTSV,CXCL10,CYFIP2,CYTH2,DBI,DBN1,DCTPP1,DDB2,DDIT4,DDX11,DHFR,DUSP2,DUSP4,DUSP5,DUSP6,DUSP7,E2F2,Ear2,EBF1,ECM1,EGR2,EIF2B4,EIF4EBP1,EMP1,ENO1,EOMES,EPCAM,F2R,FABP4,FABP5,FARP1,FAS,FASLG,FASN,FGFR1,FN1,FOS,FOXM1,FTH1,G6PD,GABARAP,GADD45B,GADD45G,GAMT,GAPDH,GATA6,GBP3,GGH,GLRX5,GLUD1,GLUL,GOT1,GOT2,GPI,GRPEL1,GSR,H2AZ1,H6PD,HAPLN1,HES1,HIF1A,HIVEP2,HLA-A,HLA-E,HMOX1,HSPB1,ID3,IDH1,IFI16, IFIT1,IFIT3,Igha,IL10,IL17RB,IL5RA,IMPA2,IQGAP2,IRF7,ITGA3,ITGAM,ITGAX,ITM2B,JUN,KHDC3L,KRT7,LAMP1,LAMP2,LDHB,LGMN,LGR5,LXN,Ly6a (includes others),LYZ,MCM6,MCM7,MGST3,MITF,MRC1,Mt1,MTBP,MTHFR,MYC,MYCT1,MYD88,MYO1C,NDRG1,NDUFS4,NIBAN1,NME2,NOP56,NOP58,OVOL1,PAM,PAX5,PCDH18,PDGFRB,Pdlim3,PDLIM7,PECAM1,PFKFB1,PFKM,PGAM1,PGK1,PKM,PLAU,PLK1,PLSCR1,PML,POLD1,POLR1B,POLR2D,PPL,PRDX2,PRDX3,PRDX4,PRKCA,Prl2c2,PYCR1,RARA,RARG,RBBP7,RHOB,RPL10,RPL6,RRM2,S100A6,SARDH,SCAMP1,SCARB1,SCEL,SDCBP,SEMA4A,SEPHS2,SIRT2,SLC16A7,SLC1A5,SLC22A4,SLC25A5,SLC2A3,SMS,SOX5,SPN,SPP1,SQOR,SRD5A1,ST3GAL3,SUCLA2,TAT,TCF3,Tcf7,TEAD2,TERT,TESPA1,TF,TFRC,TGM1,THBS1,THOP1,TIAM1,TIMM10B,TIMP2,TNFRSF12A,TNFRSF19,TNFRSF8,TNS3,TPD52,TSPAN7,TSPO,TWSG1,TXN,UGT1A6,ULBP1,VEGFC,VIM,WLS |
 Supplementary Table 1. The detailed list of DEG TF matching with the corresponding DEG in T2DM Ly6Chigh vs. T2DM Ly6Clow MC.
